# Topical TYK2 inhibitor ameliorates psoriasis‐like dermatitis via the AKT‐SP1‐NGFR‐AP1 pathway in keratinocytes

**DOI:** 10.1002/ctm2.70256

**Published:** 2025-03-04

**Authors:** Zhiqin Fang, Rundong Jiang, Yutong Wang, Wangqing Chen, Xiang Chen, Mingzhu Yin

**Affiliations:** ^1^ Department of Dermatology, Hunan Engineering Research Center of Skin Health and Disease, Hunan Key Laboratory of Skin Cancer and Psoriasis, Xiangya Hospital Central South University Changsha Hunan China; ^2^ National Engineering Research Center of Personalized Diagnostic and Therapeutic Technology Central South University Changsha Hunan China; ^3^ Department of Dermatology University of Michigan Ann Arbor Michigan USA; ^4^ Clinical Medicine Eight‐Year Program, Xiangya School of Medicine Central South University Changsha Hunan China; ^5^ Clinical Research Center, Medical Pathology Center, Cancer Early Detection and Treatment Center, Chongqing University Three Gorges Hospital Chongqing University Wanzhou Chongqing China

**Keywords:** inflammatory response, keratinocyte, psoriasis, topical treatment, TYK2 inhibitor

## Abstract

**Introduction:**

Tyrosine kinase 2 (TYK2)‐dependent cytokine signalling is integral to the pathogenesis of psoriasis. While BMS‐986165, a highly selective TYK2 inhibitor, has recently been approved for oral treatment of psoriasis, its therapeutic potential via topical application remains unexplored.

**Objectives:**

We aim to investigate the efficacy of topically applying TYK2 inhibitor in psoriasis and to elucidate the underlying mechanisms driving the therapeutic effects of this delivery approach.

**Methods:**

1.5% BMS‐986165 ointment was applied topically to the back skin of imiquimod (IMQ)‐induced psoriatic mice. To identify potential target cells influenced by the topical TYK2 inhibitor, we performed single cell RNA sequencing (scRNA‐seq) and flow cytometry on mouse lesions. The role of TYK2 in vitro was assessed by silencing its expression or administering BMS‐986165 in human keratinocytes (KCs). Mechanistic insights into TYK2 function in KCs were further investigated using RNA‐seq, dual luciferase reporter assay and ChIP‐qPCR.

**Results:**

External use of 1.5% BMS‐986165 ointment significantly ameliorated the IMQ‐induced psoriasis‐like dermatitis. Importantly, topical TYK2 inhibitor attenuated proinflammatory capability of KCs. In vitro, TYK2 inhibition suppressed the transcription of nerve growth factor receptor (NGFR) by disrupting the AKT‐SP1 signalling pathway. This impairment hindered the activation of activator protein 1 (AP1), thereby weakening the proinflammatory potential of KCs.

**Conclusion:**

This study reveals a novel therapeutic potential for selective TYK2 inhibitor in topical manner on psoriasis therapy, which might prompt the development of topical treatment for psoriasis. Crucially, our findings provide an underexplored regulatory mechanism of TYK2 inhibitor in psoriasis.

**Key points:**

- Topical TYK2 inhibitor alleviates psoriasis‐like dermatitis.
- Topical TYK2 inhibitor reduces psoriasis progression through restraining the inflammatory responses of keratinocytes.
- The inhibition of TYK2 regulates the inflammatory response of keratinocytes through AKT‐SP1‐NGFR‐AP1 pathway.

## Graphical abstract

- Topical tyrosine kinase 2 (TYK2) inhibitor alleviates psoriasis‐like dermatitis.
- Topical TYK2 inhibitor reduces psoriasis progression through restraining the inflammatory responses of keratinocytes.
- The inhibition of TYK2 regulates the inflammatory response of keratinocytes through AKT‐SP1‐NGFR‐AP1 pathway.

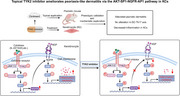

## 1 INTRODUCTION

Psoriasis is a common, chronic inflammatory skin condition characterised by aberrant interactions between hyperactivated epidermal keratinocytes (KCs) and infiltrating immune cells.^1^, ^2^, ^3^, ^4^, ^5^ The excessive and inappropriate cytokines bind to corresponding cytokine receptors and activate the downstream Janus kinase‐signal transducer and activator of transcription (JAK‐STAT) pathway, promoting the maturation of conventional dendritic cells (DCs), the differentiation of T helper (Th) cell and KC activation, thus sustaining a cutaneous self‐perpetuating inflammatory loop.^6^, ^7^, ^8^, ^9^, ^10^ As a member of JAK family, tyrosine kinase 2 (TYK2) mainly participates in the signalling transduction of type I interferons and IL‐12/IL‐23, which are pivotal in driving DC and Th cell hyperactivation in psoriasis.^7^, ^11^, ^12^, ^13^ Beyond these immune pathways, TYK2 also regulates the IL‐10 family, including IL‐19, IL‐20, IL‐22, IL‐24, all of which contribute to the inflammatory response and hyperproliferation of KCs.^12^, ^14^, ^15^, ^16^ This highlights a previously overlooked role of TYK2 in modulating KC behaviour during psoriasis pathogenesis.

Topical therapy remains a cornerstone of treatment for mild‐to‐moderate psoriasis, offering the advantage of minimising systemic risks such as hepatorenal toxicity and infections.^1^, ^17^, ^18^, ^19^ Nonetheless, the development of novel topical agents is imperative due to significant drawbacks in current medications, including local irritation, inadequate efficacy and limited options.^2^, ^20^ Recently, the oral administration of BMS‐986165 (deucravacitinib), a novel allosteric TYK2 inhibitor, has been approved for moderate‐to‐severe plaque psoriasis.^21^ However, the efficacy of topical TYK2 inhibitor in psoriasis remains unclear.

Activator protein 1 (AP1), a transcription factor composed of Fos (c‐Fos, FosB, Fra‐1, Fra‐2) and Jun (c‐Jun, JunB, JunD), plays a crucial role in inflammatory responses by forming homodimers or heterodimers with transcriptional activity.^22^, ^23^, ^24^ As functionally antagonistic members of AP‐1, JunB expression is significantly decreased in lesions from severe psoriasis patients, whereas c‐Jun is barely in normal skin but obviously elevated in psoriatic epidermis.^25^ Consistently, c‐Jun expression is increased in imiquimod (IMQ)‐induced psoriasis‐like lesions of mice.^26^ Knockout of c‐Jun or inhibition of its upstream molecule, JNK, significantly ameliorates IMQ‐induced psoriatic inflammation,^27^ suggesting JNK/c‐Jun axis as potential therapeutic targets for psoriasis. However, the mechanism underlying c‐Jun activation in psoriatic lesions is not well understood. In this study, we discover that the topical application of a selective TYK2 inhibitor markedly attenuates the IMQ‐induced psoriatic dermatitis. Unlike systemic treatments, topical TYK2 inhibition did not affect the dermal DC‐Th17 axis. Instead, it suppressed c‐Jun activation through the AKT‐SP1‐NGFR signalling pathway, resulting in decreased inflammatory responses in KCs and marked improvement in psoriasis‐like lesions.

## 2 MATERIAL AND METHODS

### 2.1 Mice

All mice were acquired from Hunan SJA Laboratory Animal Co, Ltd. To assess the phenotype stability offered by the topical formulation of BMS‐986165, experiments were conducted repeatedly using BALB/c mice aged 7–9 weeks. Both male and female mice were included without preference, ensuring gender consistency within each matched experimental and control group. The mice were housed under specific pathogen‐free conditions and maintained on a regular 12‐h light/dark cycle. Environmental conditions were tightly controlled at 22 ± 1°C with 40%–60% relative humidity.

### 2.2 IMQ‐induced psoriasis animal model and topical administration of ointment

Psoriasiform skin inflammation was induced by topically applying a cream containing 5% IMQ (cat.19080639, Sichuan Med‐Shine) to the mice back skin. The backs of the mice were shaved prior to treatment, and they were randomly assigned into three groups: Control, IMQ + Vaseline and treatment groups (IMQ + BMS‐986165 or IMQ + NGFRi). All groups, except the control group, received a daily dose of 62.5 mg of IMQ cream per mouse for five consecutive days. At least 2 h after daily IMQ application, the Vaseline group was treated with 100 mg of Vaseline ointment (composition^28^: 81% Vaseline (w), 9% Lanolin (w), 10% dimethylsulfoxide (DMSO) (v)), and the treatment group received 100 mg of Vaseline ointment containing either 1.5% BMS‐986165 (cat. S8879, Selleck) or the nerve growth factor receptor (NGFR) antagonist THX‐B (cat. HY‐137122, MCE). Disease severity was evaluated by scoring erythema, infiltration and scaling of the lesions on a scale from 0 to 4 (0 = none; 1 = slight; 2 = moderate; 3 = marked; 4 = severe). The cumulative score, representing the disease severity, ranged from 0 to 12. Additionally, the spleen index of mouse was defined as the ratio of spleen weight (mg) to body weight (g).

### 2.3 Histological analysis

Dorsal lesion skins were harvested from mice, fixed in 4% paraformaldehyde for at least 24 h, then dehydrated and embedded in paraffin. Tissue sections were then stained with haematoxylin and eosin (H&E) following standard protocols. In regard to immunofluorescence (IF) staining, sections were deparaffinised and underwent antigen retrieval before being stained with an anti‐PCNA antibody (cat. ab15497, Abcam, 1:200). After overnight incubation with anti‐PCNA at 4°C, sections were treated with an Alexa Fluor 594‐conjugated Immunoglobulin G (IgG) secondary antibody (cat. A21207, Thermo Fisher, 1:1000) for 2 h. Slides were then covered with anti‐fade mounting medium containing 4',6‐diamidino‐2‐phenylindole (cat. ab104139, Abcam) in darkness. Fluorescent Microscopy (Nikon, ECLIPSE Ts2R) was used for imaging, and Image J software for analysis of the IF staining.

### 2.4 Cell culture and stimulation

The human immortalised KC cell line HaCaT was acquired from American Type Culture Collection and grown in Roswell Park Memorial Institute (RPMI) 1640 medium supplemented with 10% foetal bovine serum (FBS), while the 293T cell was cultured in Dulbecco's Modified Eagle Medium/high glucose with 10% FBS. Primary normal human epidermal keratinocytes (NHEK) were isolated from discarded foreskin biopsy tissues collected from the Department of Urology, Xiangya Hospital, Central South University, as previously described.^29^ All cells were maintained at 37°C and 5% CO_2_.

For stimulation experiments, KCs were treated with human IL‐17A (200 ng/mL, cat. 7955‐IL‐100/CF, R&D), IL‐22 (50 ng/mL, cat. 13059‐HNAE, Sino Biological) and TNF‐α (10 ng/mL, cat. 210‐TA‐005, R&D) for 48 h. Additionally, 100 ng/mL nerve growth factor (NGF) recombinant protein (cat. 11050‐HNAC, Sino Biological), specified concentrations of BMS‐986165 (dissolved in DMSO) and Ro 08‐2750 (dissolved in DMSO, cat. HY‐108466, MCE) were used to stimulate the HaCaT cell line for 48 h.

### 2.5 Transfection experiments

HaCaT cell line was transfected with siSTAT3 (synthesised by Genepharma) or siSP1 (synthesised by Beijing Syngentech Co., Ltd.), along with a control vehicle using Lipofectamine 2000 Transfection Reagent (cat. 11668019, Thermo Fisher) at least 48 h. The targeting sequences were as follows: human siSTAT3: TTACGTCCGTTAGACAACG; human siSP1: TATTAGACAACCAAACGTG. For shRNA‐mediated knockdown or gene overexpression, 293T cells were co‐transfected with shRNA constructs targeting TYK2, NGFR or oeNGFR (all synthesised by Beijing Syngentech Co., Ltd.), along with pspAX2 and PMD2.G, and cultured overnight. After 48–72 h, the lentiviral particles were concentrated and subsequently used to transfect KCs to achieve knockdown or overexpression of target genes.

### 2.6 CCK‐8 assay

Cell proliferation was evaluated with the CCK‐8 assay (cat. B34302, Selleck). Briefly, KCs with the specific treatment were seeded in 96‐well plates at an appropriate density. Cell proliferation was monitored every 24 h by adding 10 µL of CCK‐8 solution to 100 µL of cell medium in each well. After 2 h of incubation, absorbance was measured at 450 nm.

### 2.7 Colony formation assay

To evaluate cell colony capacity, treated KCs were seeded into six‐well plates at 1500 cells per well and maintained for 1–2 weeks. The colonies were washed with Dulbecco's phosphate buffered saline (DPBS), and then fixed with 4% paraformaldehyde for 15 min, subsequently stained with Crystal Violet Staining Solution (cat. C0121, Beyotime) for 20 min. After washing with DPBS and drying, the colonies was imaged using an EPSON scanner.

### 2.8 Flow cytometry

Inguinal lymph nodes (iLN) from mice were mechanically dissociated into single‐cell suspensions, while peripheral blood mononuclear cells (PBMC) were pretreated with red blood cell (RBC) lysis buffer (cat. 555899, BD Pharmingen). Mouse back skin samples were digested overnight with RPMI 1640 medium containing 2 mg/mL Dispase II (cat. D4693, Sigma) at 4°C to separate epidermis from dermis. The dermis was then mechanically disrupted and further digested with RPMI 1640 medium containing 2 mg/mL Collagenase IV (cat. V900893, Sigma) at 37°C for 1 h, followed by filtration through 70 µm strainers. For surface staining, cells were labelled on ice with various antibodies targeting specific membrane antigens for 40 min, protected from light. Viability was assessed using viability dyes (cat. 564997, BD; cat. 423102, Biolegend). In regard to intracellular cytokine staining, cells were treated with Cell Stimulation Cocktail (cat. 00‐4975‐93, eBioscience) at 37°C for 6 h. Cells were then incubated overnight with FOXP3/Transcription Factor Staining Buffer (cat. 00‐5523‐00, eBioscience) before staining with antibodies against cytokines. The following antibodies were from Biolegend: anti‐mouse CD45 (cat. 103116), CD3 (cat. 100328), IL‐17A (cat. 506904), CD11c (cat. 117306), CD64 (cat. 139303), CD80 (cat. 104729), CD86 (cat. 105123), I‐A/I‐E (cat. 107625). The following antibodies were from BD Pharmingen: anti‐mouse TCRγ (cat. 553177), CD4 (cat. 562891), IFN‐γ (cat. 557649), IL‐23 (cat. 565317). Anti‐mouse IL‐22 (cat. 17‐7222‐82) was purchased from Thermo Fisher. Data analysis was conducted with the FlowJo v10.8.1 software.

### 2.9 RNA isolation and quantitative PCR

Fresh lesional samples were collected from mice and immediately flash‐frozen, and thoroughly homogenised. Total RNA was extracted from pretreated tissues or cultured cells using MagZol reagent (cat. R4801‐01, Magen). Next, cDNA synthesis was performed using a kit (cat. 11141ES60, Yeasen), and quantitative polymerase chain reaction (qPCR) was carried out with a kit (cat. B21703, Bimake) following the manufacturer's instructions. All primers are listed in Table S1.

### 2.10 Western blot

The protein lysates were separated by sodium dodecyl sulphate‐polyacrylamide gel electrophoresis (SDS‐PAGE) gel and subsequently transferred onto polyvinylidene fluoride membranes (cat. IPVH00010, Millipore). After blocking, membranes were incubated overnight at 4°C with specific primary antibodies. Anti‐TYK2 (cat. ab223733, Abcam, 1:100), Phospho‐TYK2 (cat. PA5‐105556, Thermo Fisher, 1:1000), glyceraldehyde‐3‐phosphate dehydrogenase (cat. 60004‐I‐Ig, Proteintech, 1:3000), STAT3 (cat. 9139S, CST, 1:1000), Phospho‐STAT3 (cat. 9145S, CST, 1:1000), NGFR (cat. ab52987, Abcam, 1:1000), SAPK/JNK (cat. 9252S, CST, 1:1000), Phospho‐SAPK/JNK (cat. 4668S, CST, 1:1000), c‐Jun (cat. ab40766, Abcam, 1:1000), Phospho‐c‐Jun (cat. 3270S, CST, 1:1000), p44/42 MAPK (Erk1/2; cat. 4695S, CST, 1:1000), Phospho‐p44/42 MAPK (Erk1/2; cat. 4370S, CST, 1:2000), p38 MAPK (cat. 8690S, CST, 1:1000), Phospho‐p38 MAPK (cat. 4511S, CST, 1:1000), Akt (cat. 4691S, CST, 1:1000), Phospho‐Akt (cat. 4056S, CST, 1:1000), SP1 (cat. 9389S, CST, 1:1000) and Phospho‐SP1(cat. ab59257, Abcam, 1:1000) were used as primary antibodies. HRP Goat Anti‐Mouse or Rabbit IgG (H + L; cat. AS003, cat. AS014, Abclonal, 1:5000) was used as secondary antibody. Image Studio was used for western blot analysis.

### 2.11 Luciferase reporter assay

Four separate fragments encompassing a 2000 bp region upstream from transcription start site (TSS) of NGFR were subcloned into the pGL3‐basic‐SV40‐hRluc dual promoter vector (Beijing Syngentech). These fragments including P1 (−2000 to −1501 bp), P2 (−1500 to −1001 bp), P3 (−1000 to −501 bp) and P4 (−500 to −1 bp). The designed diagrams were presented in Figure 6H. HaCaT cells seeded in 24‐well plates were transfected with NGFR luciferase reporter plasmid (1 µg) along with either siSP1 or a control siRNA (siNC). Promoter activity was measured with the Dual Luciferase Reporter Assay System (cat. E1910, Promega) and calculated as the ratio of firefly to Renilla luciferase activity.

### 2.12 ChIP‐qPCR

ChIP was performed using the Chromatin IP Kit (cat. 9003S, CST). Chromatin (10 µg) extracted from HaCaT cells was incubated with 5 µL of anti‐SP1 antibody (cat. 9389S, CST, 1:100) or an equivalent amount of negative control IgG overnight at 4°C. SYBR Green qPCR was conducted with primers targeting human NGFR: forward, GAGGAACAGGAACCGCAGTGG; reverse, GTGGGAAGCAGAGGCAAAGGG. ChIP signals were defined as a percentage of the total input chromatin.

### 2.13 RNA sequencing

mRNA was isolated using (dT)‐conjugated magnetic beads, and broken into short fragments. First‐strand cDNA was synthesised using random hexamer primers. Second‐strand cDNA synthesis was performed with dNTPs, DNA polymerase I and RNase H. The double‐stranded cDNA was purified and subjected to end repair, A tailing, and adapter ligation. Size selection was conducted with AMPure XP beads, and PCR‐amplified library was purified to prepare for sequencing. Differential expression analysis was performed using the DESeq R package, defining differentially expressed genes (DEGs) with a log2 fold change >1.0 and a *p*‐value <.05.

### 2.14 Single‐cell suspension preparation

Fresh mouse lesional tissues were quickly stored in Tissue Storage Solution (cat. 130‐100‐008; Miltenyi Biotec) after sacrificed, washed thrice with Hanks Balanced Salt Solution before digestion. The separated epidermis was digested into single‐cell suspensions using trypsin‐ethylene diamine tetraacertic acid, while the dermis was mechanically disrupted and treated with 2 mg/mL Collagenase IV in RPMI 1640 for 1 h at 37°C before filtration through 70 µm strainers. The combined cell suspensions from the epidermis and dermis were then treated with a kit (cat. 130‐090‐101; Miltenyi Biotec) to enhance cell viability for subsequent single‐cell sequencing.

### 2.15 Single‐cell RNA sequencing and data processing

scRNA‐seq libraries were constructed following the GEXSCOPE® Single Cell RNA Library Kits protocol (Singleron).^30^ Individual libraries were diluted, pooled and sequenced on an Illumina HiSeq X. FASTQ files were processed using SCOPE‐tools. This process involved extracting and correcting cellular barcodes, aligning reads to the GRCh38 reference genome and quantifying gene expression. The unique molecular identifier (UMI) matrix was converted to Seurat objects using Seurat package (version 4.1.0).^31^ Data filtering criteria included removing cells with UMI counts over 20 000, gene counts under 200 or mitochondrial gene proportions above 10%.

### 2.16 Cell type annotation

Seurat package was used (version 4.1.0) for downstream normalisation and clustering analysis post initial quality control. Normalisation was performed with the ‘NormaliseData’ function, and data scaling with the ‘ScaleData’ function accounted for mitochondrial and ribosomal contents. Subsequently, principal component analysis was conducted using the top 2000 most variable genes, and the top 50 principal components (PCs) were utilised for Uniform Manifold Approximation and Projection (UMAP). Cell types were annotated based on the expression of canonical marker genes: (1) keratinocyte: Krt14, Krt5, Krt10; (2) fibroblast: Col1a1, Col1a2, Dcn; (3) neutrophil: Csf3r, Ly6g, S100a9; (4) macrophage: Lyz2, Adgre1, Itgam, Mrc1; (5) DC: Itgax, H2‐Aa, H2‐Ab1; (6) fat cell: Elovl3, Elovl6; (7) smooth muscle cell: Acta2, Mylk, Cdh5, Kdr; (8) T cell: Cd3e, Cd3d, Trac, Trdc. T cells were further subclustered and annotated using the following genes: (1) γδT: Trdc, Trgc1; (2) CD8^+^ T: Cd8a, Gzmb, Gzmc, Ccl5; (3) Th17: Trac, Cd4, Ccr6; (4) IL17‐producing γδT: Trdc, Trgc2, Il17a, Il17f, Il22.

### 2.17 Gene set enrichment analysis

DEGs were identified using the Mann–Whitney *U*‐test with the Seurat ‘FindMarkers’ function. Functional annotation of the upregulated or downregulated genes was conducted using the clusterProfiler package (version 4.2.2).^32^, ^33^ Among them, Gene Ontology (GO) and Kyoto Encyclopedia of Genes and Genomes (KEGG) enrichment analyses were performed using ‘enrichGO’ and ‘enrichKEGG’, and ‘gseKEGG’ functions, with terms as significant at *p*‐value <.05.

### 2.18 Quantifications, statistical analyses and renderings

Data are presented as mean ± standard error of the mean (SEM). Statistical analyses were performed using GraphPad Prism 10, employing unpaired Student's *t*‐test, one‐ or two‐way analysis of variance (ANOVA) as appropriate. Experiments were repeated at least three times. Statistical significance thresholds were set as *p* < .05 (*p < .05, **p < .01, ***p < .001, ****p < .0001). Mechanistic diagrams were created using BioRender.com under the publication license JV27QFOXB7YQ26TABPYC.

## 3 RESULTS

### 3.1 Topical application of TYK2 inhibitor alleviated IMQ‐induced psoriasis‐like dermatitis

**FIGURE 1:**
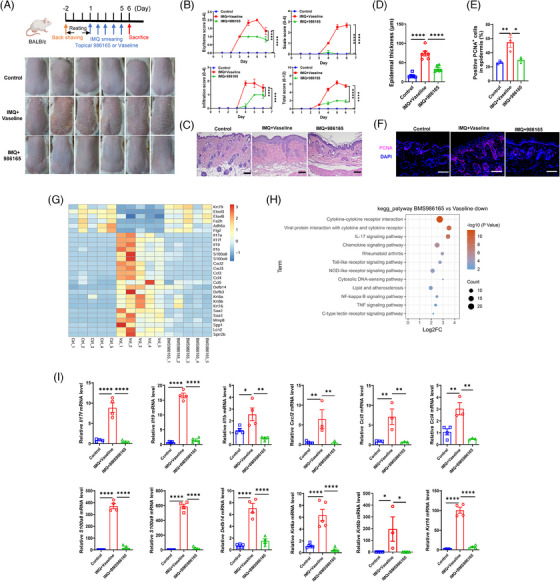
Topical application of tyrosine kinase 2 (TYK2) inhibitor alleviated imiquimod (IMQ)‐induced psoriasis‐like dermatitis. (A) (up) Experimental schedule for psoriasis mouse model topically treated with ointment. Cream containing 5% IMQ (62.5 mg/mouse) was locally applied on each mouse back skin followed by topical Vaseline or BMS‐986165 ointment (100 mg/mouse) for continuous 5 days; (down) Macroscopic images for control mice (without any treatment) and psoriatic mice either treated with Vaseline or BMS‐986165 (*n* = 6). (B) PASI score (erythema, infiltration, scale and total score; *n* = 6). (C) Representative images of haematoxylin and eosin (H&E) staining on mouse lesional skin. Scale bars, 100 µm. (D) Statistical analysis of epidermal thickness according to H&E staining (*n* = 6). (E, F) Immunofluorescent staining and statistical analysis of PCNA in mouse lesional skin (*n* = 3). Scale bars, 100 µm. (G) Heatmap of selected gene names based on transcriptomics data of mouse lesional tissues (*n* = 5). (H) Kyoto Encyclopedia of Genes and Genomes (KEGG) pathway enrichment of downregulated genes from bulk RNA‐seq in BMS‐986165‐treated skin relative to Vaseline‐treated skin (|FC| > 2, *p* < .05). (I) Quantitative polymerase chain reaction (qPCR) analysis of psoriasis‐related genes in the back skins (*n* = 3–5). *p < .05, **p < .01, ****p < .0001 by one‐way analysis of variance (ANOVA) (D, E, I) and two‐way ANOVA (B). Data are shown as mean ± standard error of the mean (SEM).

To assess the topical efficacy of the selective TYK2 inhibitor on psoriasis, we employed a preclinical mouse model induced by IMQ cream.^34^ When designing the topical formulation of the TYK2 inhibitor, we initially referred to the drug concentration of the pan‐JAK inhibitor tofacitinib (2% ointment) used in a phase 2a study for psoriasis,^35^ and also took into account the solubility of BMS‐986165 in DMSO. Eventually, we picked 1.5% as the drug concentration for in vivo experiment in order to achieve the maximum effect. And then, psoriatic mice were treated daily with 1.5% BMS‐986165 or a control ointment (Vaseline) for five consecutive days. On the sixth day, topical application of BMS‐986165 significantly reduced IMQ‐induced psoriatic symptoms such as erythema and scaling (Figure 1A), lowered the disease severity score (Figure 1B) and decreased epidermal thickness (Figure 1C,D) without affecting body weight or spleen index (Figure S1A–C). Furthermore, the proliferation marker PCNA was downregulated in the BMS‐986165‐treated group compared to the Vaseline‐treated group (Figure 1E,F). Bulk RNA‐seq of skin tissues revealed expression of several psoriasis‐related genes were substantially decreased in the BMS‐986165‐treated group, including proinflammatory cytokines (*Il17a*, *Il17f*, *Il19*, *Il1b*), chemokines (*Cxcl2*, *Cxcl3*, *Ccl3*, *Ccl4*), antimicrobial peptides (*S100a8*, *S100a9*, *Defb14*) and keratins (*Krt6a*, *Krt6b*, *Krt16*; Figures 1G and S1D).^7^, ^10^, ^16^ Pathway enrichment analysis showed that downregulated DEGs were primarily involved in proinflammatory signalling pathways such as cytokine–cytokine receptor interaction, chemokine signalling and IL‐17 signalling, while upregulated genes were enriched in the biosynthesis of unsaturated fatty acids and metabolic pathways (Figures 1H and S1E). Additionally, the qPCR experiments confirmed the reduced mRNA levels of the aforementioned psoriasis‐related genes in the BMS‐986165 group (Figure 1I). Collectively, these findings demonstrated the potent anti‐psoriatic effect of topical BMS‐986165.

### 3.2 Topical TYK2 inhibition did not impact the maturation and activation of DCs in lesional skin

Following the efficacy confirmation, we systematically investigated composition and state of respective cell types by conducting scRNA‐seq on total skin cells isolated from topical Vaseline treatment mice lesions (IMQ_Vas) and topical BMS‐986165 treatment mice lesions (IMQ_986165). After selecting variable genes and conducting UMAP dimensionality reduction, we identified eight primary cell types, including KC (*Krt14*, *Krt5*, *Krt10*), fibroblast (*Col1a1*, *Col1a2*, *Dcn*), neutrophil (*Csf3r*, *Ly6g*, *S100a9*), macrophage (*Lyz2*, *Adgre1*, *Itgam*), DC (*Itgax*, *H2‐Aa*, *H2‐Ab1*), fat cell (*Elovl3*, *Elovl6*), smooth muscle cell (*Acta2*, *Mylk*, *Cdh5*) and T cell (*Cd3e*, *Trac*, *Cd3d*, *Trdc*; Figure S2A–C). Notably, the proportion of KCs decreased significantly in the treatment group (IMQ_Vas 70.5% vs. IMQ_986165 49.2%), while the proportions of DCs and T cells showed slight changes (Figure S2D).

Given that oral TYK2 inhibitors primarily block IFN‐α and IL‐23 signalling,^36^, ^37^ we first focused on DCs (Figure S2E), and assessed whether topical BMS‐986165 affected DC maturation and activation induced by IFN‐α. Analysis of DC activation markers (*Cd40*, *H2‐Aa*, *H2‐Ab1*) and cytokine gene expression (*Il23a*) showed no significant differences between the groups (Figure S2F). In addition, we compared genes that were differentially expressed in DCs from IMQ_Vas and IMQ_986165 group (Figure S2G), and according to these DEGs, DCs from two groups did not display significant difference (*p* > .2) on antigen processing and presentation and cell migration function, suggested by gene set enrichment analysis (GSEA; Figure S2H,I). These results were further solidified by means of flow cytometry assay, in which CD86, MHC‐II and IL‐23 protein expression levels similarly remained consistent between Vaseline and BMS‐986165 group (Figures S2J,K and S3A). These findings imply that DCs are not the primary targets of this topical formulation's therapeutic effects.

### 3.3 Local TYK2 suppression limited inflammatory capacity of T cells without affecting Th17 subset

**FIGURE 2:**
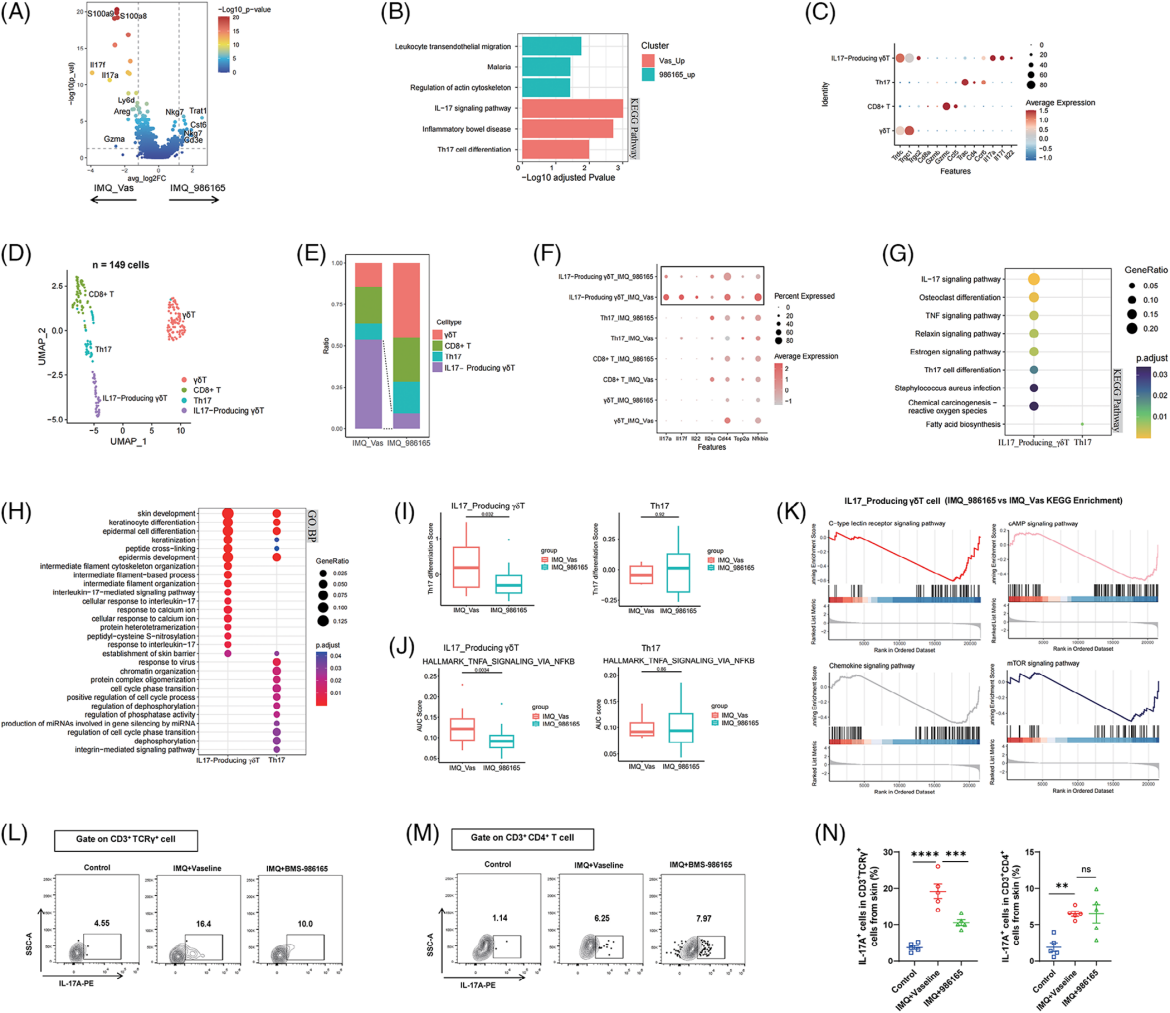
Local tyrosine kinase 2 (TYK2) suppression limited inflammatory capacity of T cells without affecting Th17 subset. (A) Volcano plot showing the differentially expressed genes (DEGs) of T cells between IMQ_986165 and IMQ_Vas. (B) The enriched Kyoto Encyclopedia of Genes and Genomes (KEGG) terms in total T cells of IMQ_986165 and IMQ_Vas. (C) Dot plot displaying the expression of marker genes in T cell subgroups. (D) Uniform Manifold Approximation and Projection (UMAP) visualisation of T cells, different colours represent distinct sub‐populations. (E) The proportion of different cell types among samples. (F) Dot plot showing *Il17a*, *Il17f*, *Il22*, *Il2ra*, *Cd44*, *Top2a* and *Nfkbia* expression comparing IMQ_986165 to IMQ_Vas in the IL17‐producing γδT, Th17, Cd8^+^ T and γδT. (G) Dot plot showing KEGG pathway enrichment on DEGs (FC <‐2, *p* < .05) in the IL17‐producing γδT and Th17. (H) Dot plot showing Gene Ontology (GO) enrichment on DEGs (FC <‐2, *p* < .05) in the IL17‐producing γδT and Th17. (I) The distribution of Th17 differentiation score in IL17‐producing γδT and Th17 cells among IMQ_Vas and IMQ_986165. (J) The AUC score of TNFA_SIGNALING_VIA_NFKB pathways in IL17‐producing γδT and Th17 cells among IMQ_Vas and IMQ_986165. (K) Enriched pathways of IL17‐producing γδT cells comparing IMQ_986165 to IMQ_Vas by gene set enrichment analysis (GSEA) analysis. (L, M) The IL17A^+^ cells in CD3^+^ TCRγ^+^ T cells (L) and CD3^+^ CD4^+^ T cells (M) of lesional dermal cells. (N) Statistic histogram (*n* = 5). Wilcoxon signed‐rank test for I and J. ns, not significant. **p < .01, ***p < .001, ****p < .0001 by one‐way analysis of variance (ANOVA) (N). Data are shown as mean ± standard error of the mean (SEM).

Next, we shifted focus to another critical signal, IL‐23, which is crucial for the differentiation and maturation of Th17 cells. scRNA‐seq analysis of total T cells from mouse lesional skin revealed the diminished enrichment of IL‐17‐related signalling pathways in the T cells from the IMQ_986165 group (Figure 2A,B), indicating a mitigated inflammatory response capability in T cells from the BMS‐986165‐treated group. Further analysis through deeper dimensional reduction identified four T cell subsets (γδT, CD8^+^ T, Th17 and IL17‐producing γδT; Figure 2C,D). Notably, the population of IL17‐producing γδT cell, rather than Th17 cell, was significantly decreased in IMQ_986165 group (Figure 2E), implying that the modulation of psoriasis outcomes by topical TYK2 inhibitor was not relied on suppressing Th17 cell function. Moreover, downregulated expression of proinflammatory genes (*Il17a*, *Il17f*, *Il22*), the activation marker *Cd44* and the transcription factor *Nfkbia* were predominantly observed in IL17‐producing γδT cells, instead of Th17 or other T cell subsets (Figure 2F). Both enrichment analysis of downregulated DEGs and enrichment scores in T cell subtypes showed that IL‐17‐related inflammatory signalling was selectively inhibited in IL17‐producing γδT cells, rather than Th17 subset (Figure 2G–J). Additionally, functional enrichment analysis revealed that the decreased DEGs in the IL17‐producing γδT cells of IMQ_986165 group overlapped with genes associated with the C‐type lectin receptor, cAMP, chemokine and mTOR signalling pathways (Figure 2K), related to T cell activation,^38^, ^39^ indicating that topical BMS‐986165 significantly weakens the inflammatory response capabilities of IL17‐producing γδT cells in lesional skin. Importantly, flow cytometry further confirmed a marked decrease in the IL‐17A^+^ TCRγ^+^ T cell population without affecting the IL‐17A^+^ CD4^+^ T subtype in the BMS‐986165‐treated group compared to Vaseline group (Figures S3B and 2L–N). Further analyses on the function of DCs and CD4^+^ T cells revealed no significant alterations in PBMC and iLN (Figure S3C–G), suggesting that the topical application of TYK2 inhibitor did not significantly affect the global immunity of psoriasis mice. These results also indicate that external use of TYK2 inhibitor could better avoid systemic adverse reactions caused by systemic TYK2 inhibition, such as upper respiratory tract infections.^21^

### 3.4 Topical TYK2 inhibition attenuated inflammatory response in lesional KCs

**FIGURE 3:**
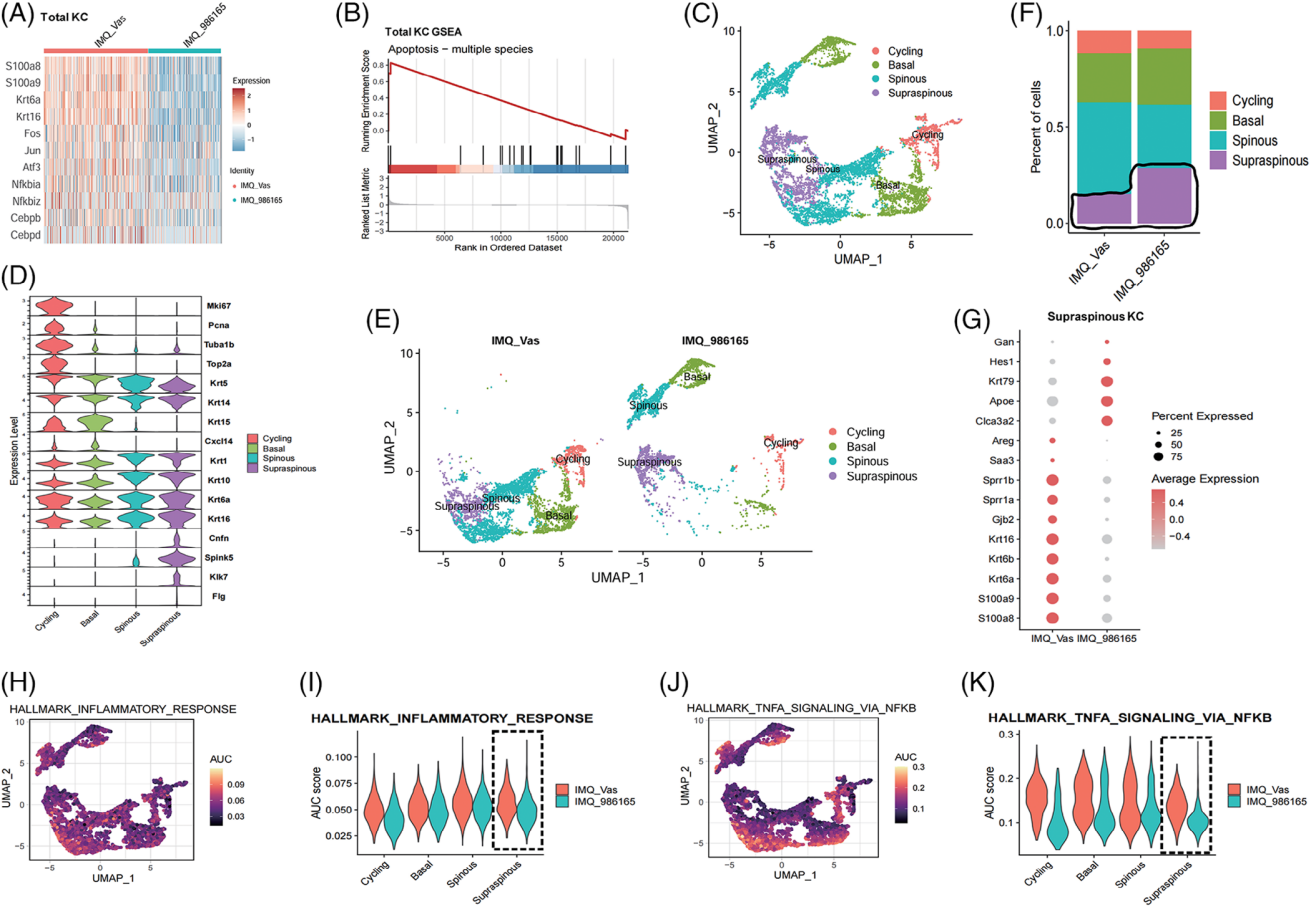
Topical tyrosine kinase 2 (TYK2) inhibition attenuated inflammatory response in lesional keratinocytes (KCs). (A) Heatmap showing the differentially expressed genes (DEGs) between IMQ_Vas and IMQ_986165 KCs. (B) Enriched pathways of total KCs by gene set enrichment analysis (GSEA) analysis. (C) Uniform Manifold Approximation and Projection (UMAP) plot of KCs. (D) Violin plot showing the commonly differentiation marker genes expression in KCs subsets. (E) UMAP plot of KCs under different treatment conditions. (F) Proportions of KCs subsets under different treatment conditions. (G) Dot plot showing the DEGs comparing IMQ_986165 to IMQ_Vas in the Supraspinous KC. (H, I) Inflammatory response pathway AUC score cluster distributions (H) and respective AUC scores in the KC subtypes (I), each subtype is split by treatment conditions. (J, K) TNFA_SIGNALING_VIA_NFKB KRAS pathway AUC score cluster distributions (J) and respective AUC scores in the KC subtypes (K), and each subtype is split by treatment conditions.

After analysing DC and Th17 cell functionality, we turned focus to the KC sub‐population, which plays a key role in the pathogenesis of psoriasis (Figure S4A). Initially, scRNA‐seq on the overall KC population showed significant reductions in psoriasis‐related genes such as S100 molecules, keratins and transcription factors like *Nfkbia*, *Nfkbiz*, *Cebpb* and *Cebpd* in the treatment group (Figure 3A). Moreover, GSEA analysis suggested increased apoptosis process in the treatment group's KCs (Figure 3B), which aligns with the anti‐psoriatic phenotype observed with the topical application of BMS‐986165. Through UMAP dimensionality reduction of 9249 KCs, we obtained 14 clusters, further categorised into four subtypes: Cycling, Basal, Spinous and Supraspinous, based on established molecular markers (Figures S4B and 3C,D).^40^, ^41^ Notably, the distribution of KCs was different between the IMQ_986165 group and the IMQ_Vas group (Figure 3E). The pathogenesis of psoriasis involves incomplete differentiation of KCs, with a reduction in cells in the late differentiation stage (supraspinous layer).^5^ However, it was markedly improved by topical TYK2 inhibition, as demonstrated by an increased proportion of the Supraspinous subtype (Figure 3F). Additionally, gene expression in this subtype got normalised post‐BMS‐986165 treatment; for example, the gene *Hes1*, typically downregulated in psoriatic lesions,^42^ was significantly increased, whereas antimicrobial peptides and keratins were substantially reduced in the treatment group (Figure 3G). Further analysis revealed that the alleviated inflammatory response in KCs, particularly in the Supraspinous subtype, was significantly mitigated compared to the Vaseline group (Figure 3H–K). Given that the Supraspinous subtype is located in the outermost skin layer and is more likely to get affected by topical treatments, these findings suggest that topical TYK2 inhibition may alleviate KC‐driven inflammation by specifically targeting this subtype, thereby improving the phenotype of psoriatic mice.

### 3.5 TYK2 inhibition suppressed the proliferation and inflammation of KCs in vitro

**FIGURE 4:**
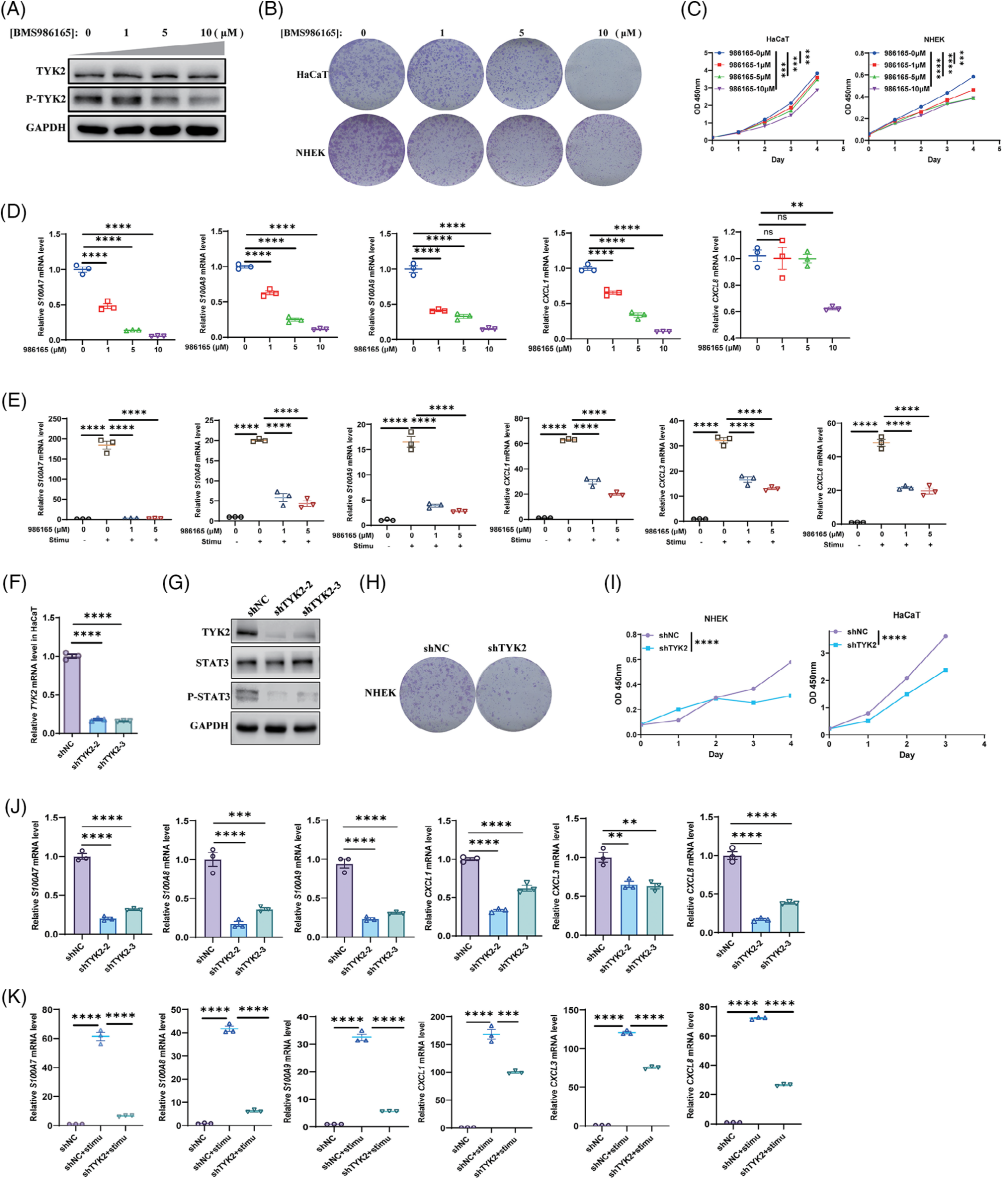
Tyrosine kinase 2 (TYK2) inhibition suppressed the proliferation and inflammation of keratinocytes (KCs) in vitro. (A) Western blot analysis of phosphorylation and total protein expression of TYK2 in HaCaT cells after treated with BMS‐986165 for 48 h. (B) Colony formation assay of HaCaT (up) and normal human epidermal keratinocytes (NHEK) (down) cells after treatment with BMS‐986165. (C) The proliferative ability of HaCaT (left, *n* = 5) or NHEK (right, *n* = 4) cells treated with a serial dose of BMS‐986165 was evaluated at different time period by CCK‐8 assay. (D, E) Quantitative polymerase chain reaction (qPCR) analysis of several psoriasis‐associated antimicrobial peptides and chemokines in HaCaT cells after treatment with BMS‐986165 for 48 h in absence (D) or presence (E) of stimulation of cytokines (200 ng/mL IL‐17A, 50 ng/mL IL‐22 and 10 ng/mL TNF‐α; *n* = 3). (F) qPCR analysis of the efficiency of knockdown on two shRNA sequences (shTYK2‐2 and shTYK2‐3) targeting TYK2 in HaCaT cell line (*n* = 3). (G) Western blot analysis of specified protein expression in HaCaT cells transfected stably with two different shRNA sequences targeting TYK2. (H) Colony formation assay of NHEK cells treated with or without TYK2 silencing (shTYK2‐2 was used). (I) The proliferative ability of NHEK (left, *n* = 5) or HaCaT (right, *n* = 3) cells after TYK2 knockdown (shTYK2‐2 was used) was evaluated by CCK‐8 assay. (J, K) qPCR analysis of several psoriasis‐associated proinflammatory mediators in HaCaT cells after TYK2 silencing (shTYK2‐2 was used) in absence (J) or presence (K) of cytokine stimulation (IL‐17A, IL‐22 and TNF‐α; *n* = 3). ns, not significant. **p < .01, ***p < .001, ****p < .0001 by one‐way analysis of variance (ANOVA) (D–F, J, K) and two‐way ANOVA (C and I). Data are shown as mean ± standard error of the mean (SEM).

To further elucidate the role of TYK2 in KC biology, we treated human KCs with BMS‐986165 and conducted TYK2 knockdown in vitro (Figure 4A,F,G). The anti‐proliferative effects of TYK2 inhibition were demonstrated in primary KCs (NHEK) or HaCaT cell line via colony formation assays (Figure 4B,H). Additionally, CCK‐8 assays indicated that BMS‐986165 dose‐dependent treatments and TYK2 silencing in HaCaT or NHEK cells consistently reduced proliferative capacity of KCs (Figure 4C,I). Moreover, TYK2 inhibition also led to significant anti‐inflammatory effects in HaCaT cells with or without cytokine stimulation (Figure 4D,E). Notably, IL‐22 signalling transduction, which relies on TYK2, not only promotes KCs proliferation, but also enhances antimicrobial peptide production.^43^, ^44^ Therefore, HaCaT cells were initially treated with IL‐22 recombinant protein in combination with BMS‐986165 for 48 h. We found that IL‐22 alone modestly increased the expression of the S100 molecules without significantly affecting chemokines levels (Figure S5A), consistent with previous reports.^44^ On the contrary, BMS‐986165 continued to suppress the transcription of these proinflammatory genes in a concentration‐dependent manner (Figure S5A). Next, to better simulate the inflammatory cascade characteristic of psoriasis, we stimulated KCs with a combination of IL‐22, IL‐17A and TNF‐α, known to synergistically enhance the proinflammatory effects of IL‐22 in KCs.^45^ After 48 h, this cytokine trio significantly upregulated the expression of inflammatory mediators. By great contrast, TYK2 inhibitor effectively downregulated the transcription of these inflammatory genes in a dose‐dependent manner (Figure 4E). Furthermore, silencing TYK2 expression with shRNA also suppressed the production of proinflammatory mediators in HaCaT cells, both at baseline and under cytokine‐stimulated conditions (Figure 4J,K). These results provide a stronger theoretical foundation for the use of topical TYK2 inhibitor in ameliorating the phenotype of psoriasis‐like mice.

### 3.6 The anti‐inflammatory effects on KCs mediated by TYK2 inhibition were not relied solely on TYK2‐STAT3 axis

**FIGURE 5:**
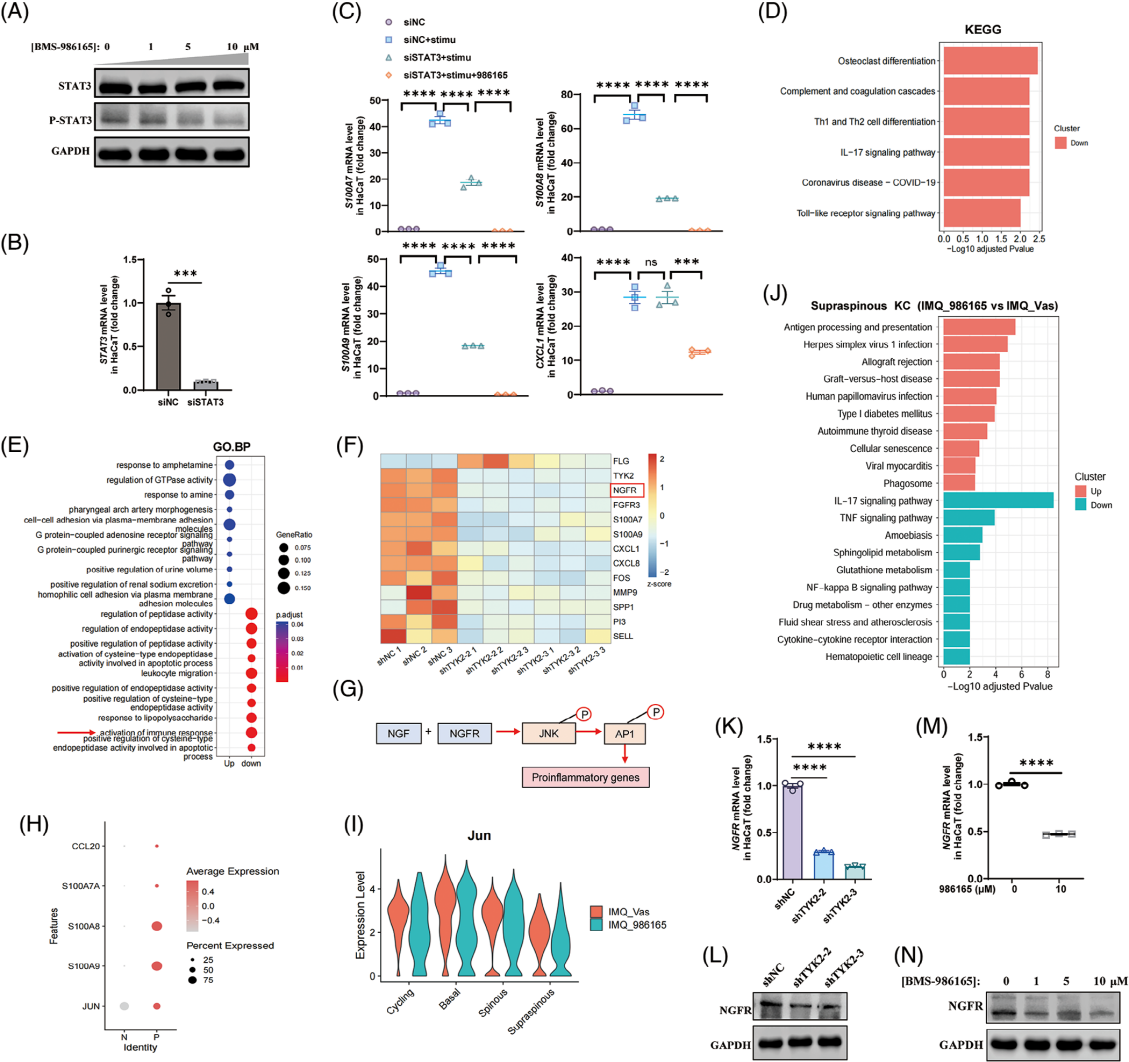
The anti‐inflammatory effect on keratinocytes (KCs) mediated by tyrosine kinase 2 (TYK2) inhibition was not relied solely on TYK2‐STAT3 axis. (A) Western blot analysis of specified protein expression in HaCaT cells given BMS‐986165 treatment for 48 h. (B) Quantitative polymerase chain reaction (qPCR) analysis of STAT3 in HaCaT cells after transient transfection with siSTAT3 for 48 h (*n* = 3). (C) qPCR of *S100A7*, *S100A8*, *S100A9* and *CXCL1* in HaCaT cells under cytokine stimulation (IL‐17A, IL‐22 and TNF‐α) after indicated treatment for 48 h (*n* = 3). (D) Kyoto Encyclopedia of Genes and Genomes (KEGG) pathway enrichment of downregulated genes from bulk RNA‐seq in shTYK2 HaCaT relative to shNC HaCaT (|FC| > 2, *p* < .05). (E) Dot plot showing Gene Ontology (GO) biological process terms enriched in upregulated and downregulated genes from bulk RNA‐seq in shTYK2 HaCaT relative to shNC HaCaT (|FC| > 2, *p* < .05). (F) Heatmap of selected gene names based on bulk RNA‐seq in HaCaT cells (*n* = 3). (G) Schematic diagram of signalling pathway that the binding of NGF to nerve growth factor receptor (NGFR) results in the downstream phosphorylation of JNK‐AP1 pathway and proinflammatory genes generation. (H) Dot plot showing the expressions of *JUN* and psoriasis‐related genes in KCs from psoriasis lesions (P) and healthy tissues (N) based on the published scRNA‐seq data (GSE 150672). (I) Violin plots show the expression of *Jun* between IMQ_Vas and IMQ_986165 in each stage of KCs. (J) KEGG analysis of upregulated or downregulated genes enriched in BMS‐986165‐treated group relative to Vaseline‐treated group according to the scRNA‐seq results on Supraspinous KCs. (K, L) qPCR (K) (*n* = 3) and western blot (L) analysis on the expression of NGFR in HaCaT cells after TYK2 knockdown. (M, N) qPCR (M) (*n* = 3) and western blot (N) analysis on the expression of NGFR in HaCaT cells after treatment with BMS‐986165 for 48 h. ns, not significant. ***p < .001, ****p < .0001 by two‐tailed unpaired *t*‐test (B, M) and one‐way analysis of variance (ANOVA) (C, K). Data are shown as mean ± standard error of the mean (SEM).

Next, we explored how TYK2 specifically regulates the function of KCs. Firstly, we investigated whether TYK2 regulates KCs by downstream STAT3, as the prevailing view that cytokines signalling through TYK2, such as IL‐22, IL‐20, IL‐24 and IL‐19, primarily target KCs and modulate cell proliferation and inflammation through the phosphorylation of STAT3.^46^ As expected, western blot results showed significant decreases of STAT3 phosphorylation mediated by TYK2 inhibition (Figures 4G and 5A). Subsequently, STAT3 silenced in HaCaT cells effectively suppressed the production of antimicrobial peptides induced by cytokines stimulation (IL‐22, IL‐17A and TNF‐α) in KCs, while the production of proinflammatory chemokine *CXCL1* remained unchanged (Figure 5B,C). Interestingly, the treatments in combination with BMS‐986165 had an additional and striking restraining effect on inflammatory genes production, hinting other potential pathways regulated by TYK2 may exist in the inflammatory process of KC except for the classical TYK2‐STAT3 axis.

Thus, we then performed RNA‐seq on TYK2‐knockdown HaCaT cells to figure out the additional regulation pathway. Consistently, downregulated DEGs mainly enriched in inflammation‐associated pathways, including IL‐17 signalling pathway and activation of immune response based on KEGG and GO enrichment (Figure 5D,E). Surprisingly, we discovered a remarkable downregulated gene called *NGFR* in both of two sequences of TYK2 silencing (Figure 5F). NGFR, as one of the NGF receptors, is well established to bind NGF, leading to the activation of JNK, a key component of the MAPK pathway and a critical mediator of the proinflammatory response.^47^ JNK consequently activates the downstream transcription factor AP1, playing key role in production of psoriasis‐associated proinflammatory genes, such as *CXCL1*, *CXCL8*, *CCL20* ^7^, ^48^ (Figure 5G). Meanwhile, we analysed the published scRNA‐seq data (GSE 150672) of KCs from psoriasis lesions and healthy tissues and found out a notable elevated expression of *JUN* (gene name of c‐Jun), an AP1 subunit, along with several increased expression of antimicrobial peptides and chemokine *CCL20* in psoriatic KCs (Figure 5H), indicating a positive correlation between *JUN* expression and the inflammatory mediators in KCs within psoriasis. Furthermore, according to scRNA‐seq data from psoriatic mouse lesions, we observed a decrease in *Jun* expression in the Supraspinous subtype of KCs in the treatment group (Figure 5I). Additionally, KEGG enrichment analysis suggested that treatment with BMS‐986165 suppressed inflammatory pathways including IL‐17 signalling and cytokine–cytokine receptor interaction pathways (Figure 5J). Therefore, we proposed a hypothesis that the anti‐inflammatory effects of BMS‐986165 in KCs might be mediated through the NGFR‐JNK‐AP1 pathway. To test this, we examined whether TYK2 modulates the expression of NGFR in KCs. Impressively, qPCR and western blot experiments validated that TYK2 inhibition by BMS‐986165 and gene silencing equally caused lower mRNA and protein expression level of NGFR in HaCaT cells (Figure 5K–N).

### 3.7 TYK2 inhibitor modulated AP1 function through the AKT‐SP1‐NGFR axis to reduce psoriatic inflammation

**FIGURE 6:**
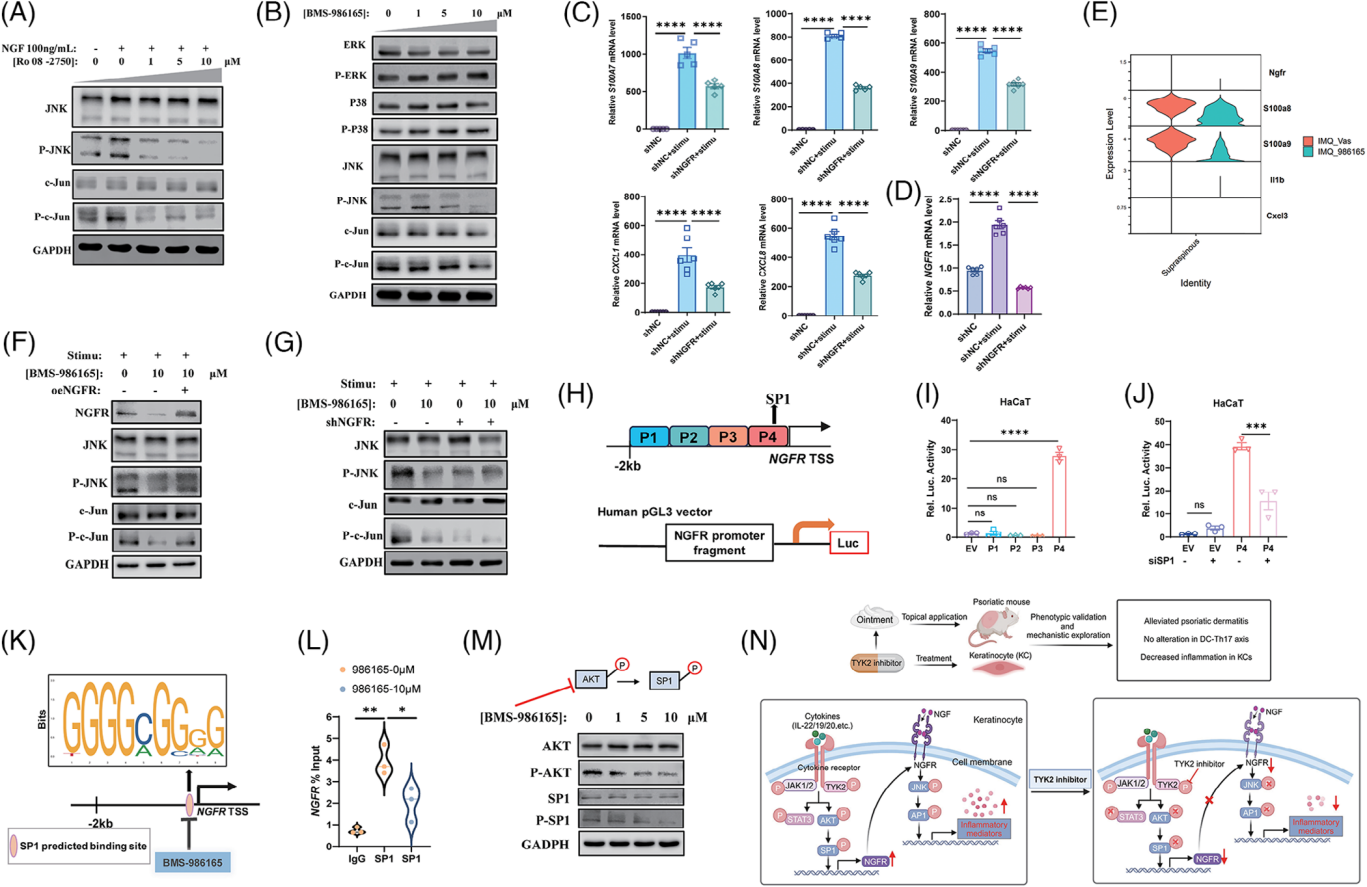
The tyrosine kinase 2 (TYK2) inhibitor reduced the generation of proinflammatory genes in keratinocytes (KCs) through AKT‐SP1‐NGFR‐AP1 pathway. (A) Western blot analysis of phosphorylation and total protein expression of JNK and c‐Jun in HaCaT cells given specified treatments for 48 h. (B) Western blot analysis of phosphorylation and total protein expression of MAPK pathway (including ERK, P38 and JNK) and c‐Jun in HaCaT cells given specified treatments for 48 h. (C, D) Quantitative polymerase chain reaction (qPCR) analysis on mRNA expression of several proinflammatory mediators (C) and *NGFR* (D) in HaCaT cells transfected with or without shNGFR (the second sequence of shNGFR) in combination with cytokines (IL‐17A, IL‐22 and TNF‐α) stimulation for 48 h (*n* = 5–6). (E) Violin plots show the expression of *Ngfr* along with several proinflammatory genes in the Supraspinous KCs. (F, G) Western blot analysis on the expression of specified proteins in HaCaT cells after different treatments for 48 h. Stimu: stimulation with cytokines IL‐17A, IL‐22 and TNF‐α. (H) Schematic diagram of nerve growth factor receptor (NGFR) promoter reporter plasmid construction. (I) Luciferase activity in HaCaT cells either transfected the empty (EV) or respective promoter reporter plasmid of NGFR (*n* = 3). (J) Luciferase activity in HaCaT cells transfected with or without SP1 siRNA in combination with the specified promoter reporter plasmid of NGFR (*n* = 3). (K) SP1‐binding site in NGFR promoter predicted by JASPAR. (L) Relative enrichment fold of NGFR promoter chromatin immunoprecipitated with SP1 in HaCaT treated with or without BMS‐986165 for 48 h analysed by qPCR (*n* = 3). (M) Western blot analysis of the phosphorylation and total protein expression of AKT and SP1 in HaCaT cells after treated with BMS‐986165 for 48 h. (N) The diagram of regulatory mechanism of topical TYK2 inhibitor in psoriasis mouse model. ns, not significant. *p <  .05, **p <  .01, ***p < .001, ****p < .0001 by one‐way analysis of variance (ANOVA) (C, D, I, J, L). Data are shown as mean ± standard error of the mean (SEM).

We next investigated whether the NGFR‐JNK‐AP1 pathway exists in KCs. In vitro, HaCaT cells were stimulated with NGF recombinant protein, both with or without Ro 08‐2750, an inhibitor that prevents NGF from binding to NGFR. Western blot analysis showed that NGF stimulation significantly enhanced the phosphorylation of JNK and c‐Jun, while Ro 08‐2750 treatment notably reduced the phosphorylation in a dose‐dependent manner (Figure 6A). Furthermore, BMS‐986165 treatment disrupted the activation of JNK and c‐Jun but did not affect other MAPK pathway members such as ERK, P38 (Figure 6B). Meanwhile, the expression of NGFR and the activation of JNK‐c‐Jun axis were also inhibited in the primary KCs after treatment with BMS‐986165 (Figure S6A). We then established an NGFR‐knockdown HaCaT cell line to assess NGFR's regulatory effect on KCs (Figure S6B,C). The knockdown of NGFR did not impact HaCaT cell proliferation (Figure S6D,E). However, it effectively inhibited the production of proinflammatory mediators when cells were stimulated with IL‐22, IL‐17A and TNF‐α (Figure 6C). Interestingly, stimulation with cytokines significantly increased NGFR transcription (Figure 6D), supporting previous findings that inhibiting TYK2 reduces the NGFR transcription. Simultaneously, reanalysis of scRNA‐seq data from psoriatic mouse skin showed that topical application of BMS‐986165 reduced NGFR expression in the Supraspinous KC subtype, accompanied by reductions in inflammatory genes such as *S100a8*, *S100a9*, *Il1b* and *Cxcl3* (Figure 6E), suggesting that TYK2 inhibitor mitigates psoriasis‐like inflammation by modulating NGFR transcription and regulating the inflammatory response in KCs. Moreover, in cytokine‐stimulated HaCaT cells, overexpression of NGFR rescued the decrease in JNK and c‐Jun phosphorylation caused by BMS‐986165 (Figure 6F), while synergistic inhibitory effect on JNK and c‐Jun activation was not detected in the HaCaT cells after dual inhibition of NGFR and TYK2 (Figure 6G), implying that BMS‐986165 primarily regulates JNK‐AP1 pathway activation through NGFR.

To elucidate the mechanism by which TYK2 inhibitor downregulates NGFR expression, based on transcription factor predictions and literature review,^49^ we hypothesised that the transcription factor SP1 binds near the TSS of NGFR, thereby regulating its expression. To test this, we constructed four NGFR reporter plasmids within the 2000 bp upstream of NGFR's TSS (Figure 6H). Dual‐luciferase reporter assays revealed that the −500 bp to −1 bp fragment (P4 fragment) exhibited significant transcriptional activity, and silencing SP1 substantially decreased NGFR transcription (Figure 6I,J). Additionally, predicted SP1 binding sites within this 500 bp segment were further validated using ChIP‐qPCR, confirming that TYK2 inhibition disrupts the binding of SP1 to the NGFR promoter, thus modulating its transcription (Figure 6K,L). Previous research indicates that the phosphorylation of AKT causes SP1 phosphorylation, thereby activating SP1 to regulate downstream target genes.^50^, ^51^ Considering that several cytokine signalling can directly induce AKT phosphorylation via TYK2 activation,^52^ we posited that inhibiting TYK2 phosphorylation would similarly diminish AKT phosphorylation, consequently curtailing SP1 activation. Western blot results demonstrated that intervention with BMS‐986165 decreased the phosphorylation levels of AKT and SP1 in HaCaT cells with dose dependence, without altering total protein (Figure 6M), illustrating that TYK2 inhibitor mainly achieve transcriptional repression of NGFR through the inactivation of SP1. Moreover, in vivo experiments substantiated the pivotal role of NGFR in the mouse model of psoriasis. Topical application of a 1.5% NGFR antagonist, THX‐B ointment (IMQ + NGFRi group), effectively mitigated IMQ‐induced psoriatic alterations in mice (Figure S7A–E), without impacting the spleen or body weight of mice (Figure S7F–H). These in vivo results, coupled with our in vitro findings, demonstrate that TYK2 inhibitor alleviates inflammation in KCs predominantly through the AKT‐SP1‐NGFR‐AP1 signalling pathway, thereby effectively reducing psoriasis‐like skin inflammation (Figure 6N).

## 4 DISCUSSION AND CONCLUSION

Topical therapy, as the major treatment for mild‐to‐moderate psoriasis, offers rapid and convenient remission of localised lesions. Importantly, for generalised skin lesions, combining topical treatments can relieve a range of adverse events associated with systemic therapies.^1^, ^53^ Therefore, the development of safe and effective novel anti‐psoriasis topical drugs holds clinical importance and substantial market potential.

JAK inhibitors, with their small molecular weight and excellent epidermal permeability, are well suited for topical application, enhancing localised efficacy and minimising systemic application‐induced adverse reactions. For instance, the JAK1/JAK2 inhibitor Ruxolitinib cream has been approved for vitiligo and atopic dermatitis.^54^ In psoriasis, topical JAK inhibitors have shown potential, albeit with varying degrees of efficacy. A phase 2a trial highlighted marginally improved outcomes with tofacitinib ointment in chronic plaque psoriasis compared to the controls,^35^ and Ruxolitinib cream at doses of 1% once daily or 1.5% twice daily significantly ameliorated psoriatic lesions.^55^ These findings indicate the significance of deeper research on topical JAK inhibitors in psoriasis. Unlike traditional pan‐JAK inhibitors and non‐selective TYK2 inhibitors, BMS‐986165 selectively targets the pseudokinase domain of TYK2, specifically inhibiting TYK2‐mediated proinflammatory signalling, which results in improved therapeutic efficacy in autoimmune disorders with fewer side effects.^21^ Based on this, we hypothesised that topical TYK2 inhibition could similarly alleviate psoriatic symptoms. Indeed, our preclinical study demonstrated that external use of TYK2 inhibitor remarkably ameliorated IMQ‐induced psoriasis‐like skin inflammation without impairing the growth or general health of the mice, representing a promising candidate for topical treatment of psoriasis. However, the long‐term efficacy, safety profile and optimal therapeutic dose of topical TYK2 inhibitor in human psoriasis remain to be further evaluated in subsequent clinical trials.

Orally administration of BMS‐986165 is recognised for its effectiveness against psoriasis, primarily by blocking type I IFN and IL‐12/IL‐23 signalling, thereby disrupting the DC‐Th17 crosstalk axis.^21^, ^36^ Surprisingly, in our study, topical application of the TYK2 inhibitor did not affect the inflammatory response in DCs or Th17 cells in the inflamed skin, iLN or PBMC, as evidenced by scRNA‐seq and flow cytometry. Instead, it obviously reduced the secretion of proinflammatory cytokines in γδT cells within psoriatic lesions. Previous studies confirmed that γδT cells are crucial in mouse models of psoriasis, while the predominant pathogenic T cells in human lesions are αβT cells.^34^ This discrepancy raises questions about whether the observed effects are specific to animal models or topical TYK2 inhibitors possess unique regulatory mechanisms affecting γδT cells, meriting further investigation in human samples.

KC, as the most superficial cell type in skin tissues, undoubtedly stays on the front line to be manipulated by topical treatment. Meanwhile, KC is the victim of multiple cytokines secreted by infiltrated immune cell and this incited KC further releases extensive proinflammatory mediators to recruit additional immune cells owning intensive inflammation response, in which a self‐perpetuating inflammatory loop forms in the skin tissue during psoriasis pathogenesis.^56^ Therefore, the mechanism of how TYK2 inhibitor acts on KC deserve profound investigation. In this study, based on extensive in vivo and in vitro data, we identified that TYK2 inhibitor directly restrains the proinflammation effects of KC, thereby ameliorating the psoriasis‐like dermatitis. Mechanistically, we discovered that TYK2 knockdown in KCs reduces the expression of several growth factor receptors, including FGFR3 and NGFR (Figure 5F). While the loss of FGFR3 in KCs does not impact skin homeostasis,^57^ NGFR is associated with the inflammatory response as an upstream regulator of the JNK‐AP1 pathway.^27^, ^47^ Thus, focusing on NGFR, we explored how TYK2 inhibition mitigates the inflammatory status of KCs. Our findings reveal that TYK2 inhibition directly suppresses AKT phosphorylation, consistent with previous report indicating that TYK2 activation triggers the PI3K‐AKT pathway.^52^ Reduced AKT phosphorylation subsequently decreases the activity of the transcription factor SP1. Notably, research of Huang et al. ulteriorly propped up our finding that phosphorylated AKT can induce SP1 phosphorylation.^50^ Utilising dual luciferase reporter assays and ChIP‐qPCR, we further identified that TYK2 inhibitor impedes SP1 binding to the NGFR promoter, leading to transcriptional downregulation of NGFR and suppression of the JNK‐AP1 pathway, thereby reducing the production of proinflammatory mediators in KC.

In summary, this study adds a promising option to the list of topical anti‐psoriasis drugs and unveils a novel regulatory mechanism for the external use of allosteric TYK2 inhibitor in treating psoriasis.

## AUTHOR CONTRIBUTIONS

**Zhiqin Fang**: Conceptualisation, Investigation, Validation, Visualisation, Writing — Original draft preparation. **Rundong Jiang**: Conceptualisation, Investigation, Software, Visualisation, Writing — Original draft preparation. **Yutong Wang**: Investigation, Visualisation, Writing — Original draft preparation. **Wangqing Chen**: Funding Acquisition, Writing — Original draft preparation. **Xiang Chen**: Funding Acquisition, Resources, Supervision, Writing — Reviewing and Editing. **Mingzhu Yin**: Conceptualisation, Funding Acquisition, Resources, Supervision, Writing — Reviewing and Editing.

## CONFLICT OF INTEREST STATEMENT

The authors declare no conflicts of interest.

## ETHICS STATEMENT

All animal care and experimental methods were approved by the Animal Care and Use Committee of the Department of Laboratory Animals at Central South University, Changsha, China (approval number CSU‐2022‐0027).

## Supporting information



Supporting information

## Data Availability

The data underlying this article will be shared on reasonable request from the corresponding author.
