# Supplementary material for: Topical TYK2 inhibitor ameliorates psoriasis‐like dermatitis via the AKT‐SP1‐NGFR‐AP1 pathway in keratinocytes
Source: Clin Transl Med. 2025 Mar 4;15(3):e70256. doi: 10.1002/ctm2.70256 (PMC11879890; doi:10.1002/ctm2.70256)
Supplement: Supplementary file 1 — Supporting information [file CTM2-15-e70256-s001.docx]

**aSupplementary Material**

**Supplemental figures and figure legends**

**
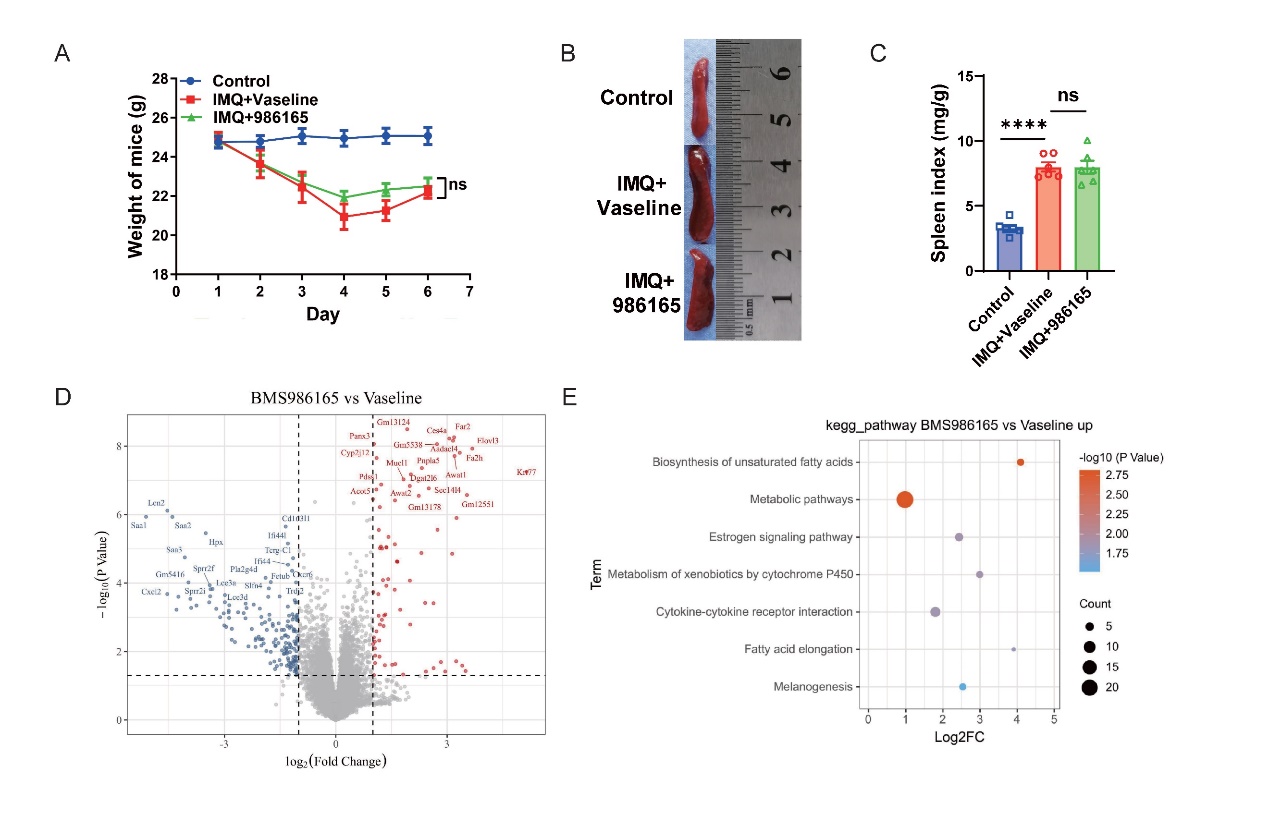
**

**Figure S1. Topical application of TYK2 inhibitor did not affect the body weight and spleen index of mice. (A)** The body weight change of mice during psoriasis model induction (n=6). **(B)** The macroscopic images of spleen. **(C)** The spleen index (n=6). **(D)** Volcano plot of significant DEGs in BMS-986165-treated mice vs Vaseline-treated mice according to bulk RNA-seq on back skin lesions. **(E)** KEGG pathway enrichment of upregulated genes from bulk RNA-seq in BMS-986165-treated skin relative to Vaseline-treated skin (|FC|>2, p<0.05). ns, not significant. ****P < 0.0001 by two-way ANOVA (A) and one-way ANOVA (C). Data are shown as mean ± SEM.

**
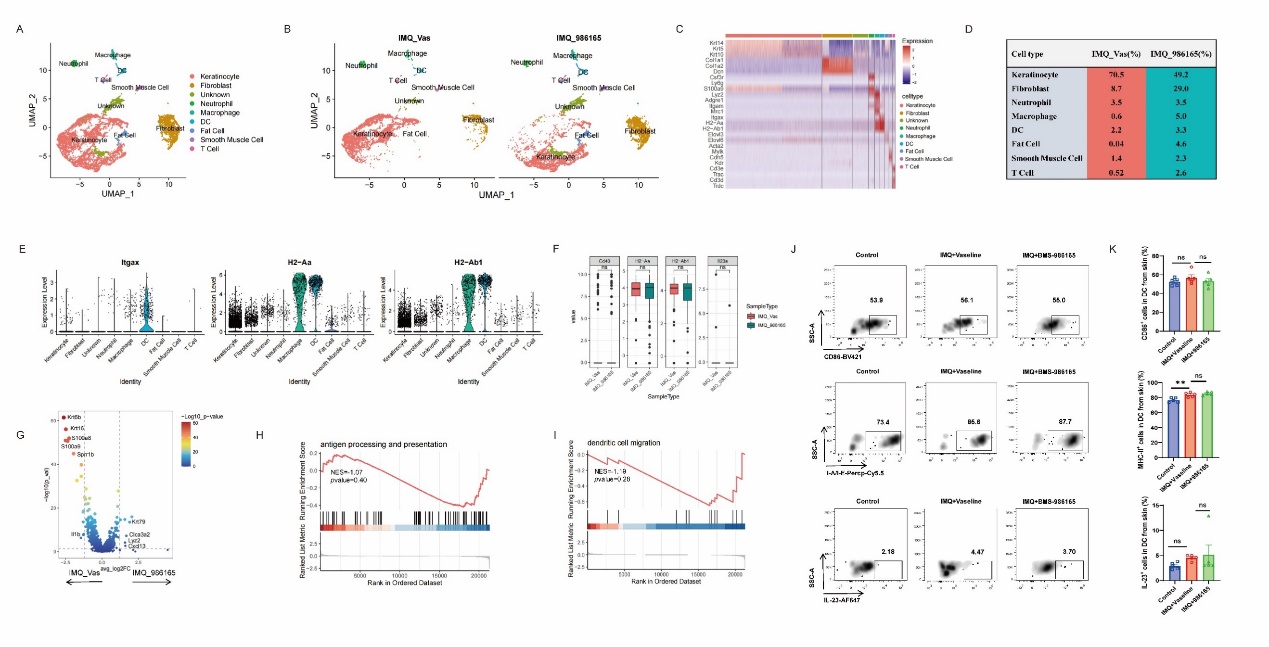
**

**Figure. S2 The proinflammatory function of dermal DC was not suppressed by topical TYK2 inhibitor. (A)** UMAP plot showing 16038 cells (IMQ_Vas and IMQ_986165 skin) colored by different cell types. **(B)** UMAP plot showing the cells colored by treatment conditions. IMQ_Vas, topical Vaseline treatment. IMQ_986165, topical BMS-986165 treatment. **(C)** Heatmap showing the representative marker genes for each cell type. The color scale represents the scaled expression of each gene. **(D)** The cell types population comparisons between IMQ_Vas and IMQ_986165. **(E)** Violin plot showing the DC markers expression in different cell types. **(F)** Gene expression level of *Cd40*, *H2-Aa*, *H2-Ab1* and *Il23a* across IMQ_Vas and IMQ_986165. **(G)** Volcano plot showing the DEGs of DCs between IMQ_Vas and IMQ_986165. **(H, I)** Enriched pathways of DCs by GSEA analysis. **(J)** The proportion of CD86^+^ cells, I-A/I-E^+^ cells, and IL23^+^ cells in DC cells among diverse treatment group detected by flow cytometry. **(K**) Histogram of the proportion of CD86^+^, I-A/I-E^+^ and IL23^+^ in dermal DC cells (n=5). ns, not significant. **P < 0.01 by Wilcoxon signed-rank test (F) and one-way ANOVA (K). Data are shown as mean ± SEM.

**
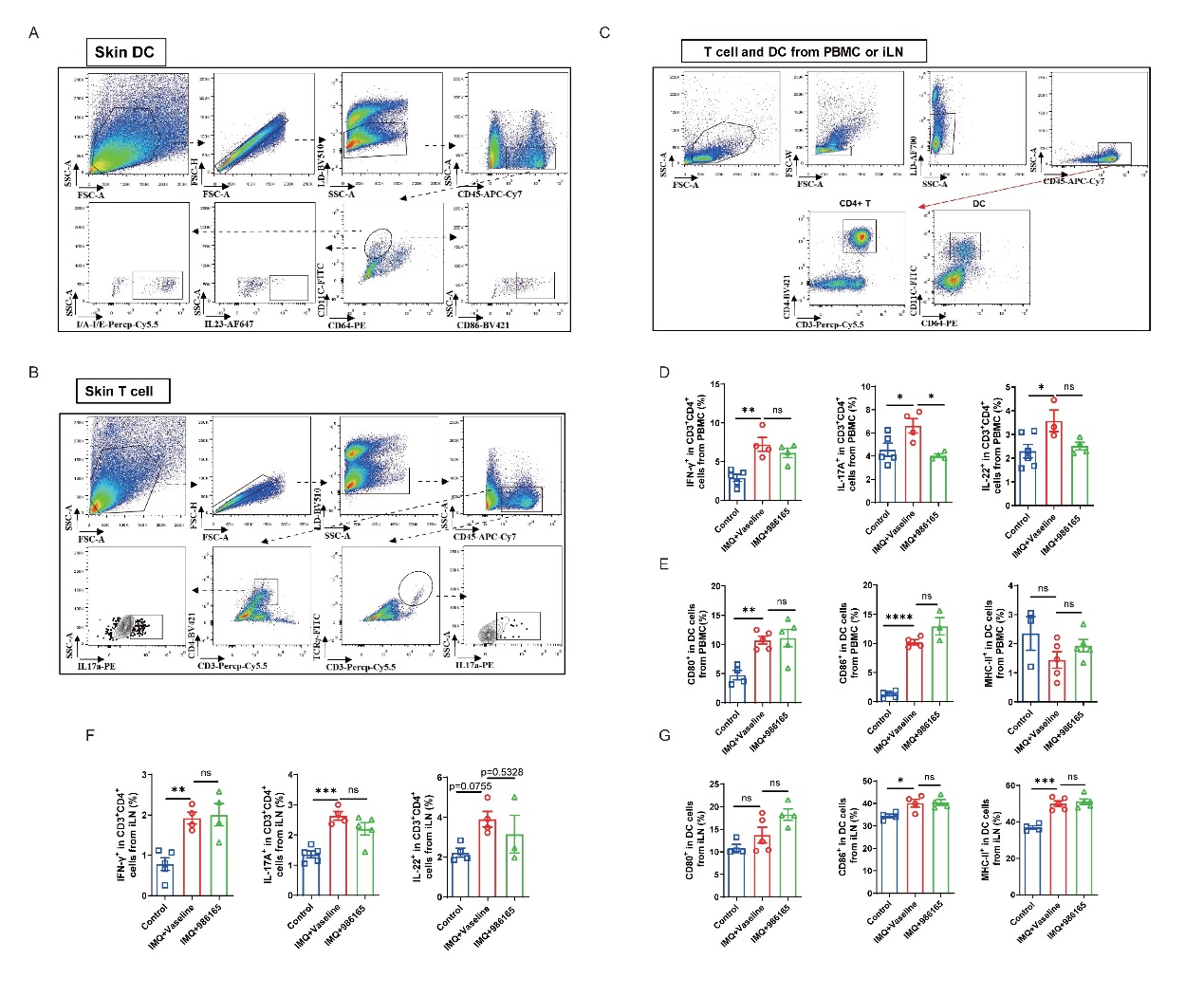
**

**Figure S3. The topical TYK2 inhibition had no suppression on function of DC and T cells of PBMC and iLN. (A,** **B)** Gating strategies of DC **(A)** and T cells **(B)** from mouse back skin. **(C)** Gating strategies of DC and T cells from PBMC or iLN. (**D, E)** Flow cytometry quantification of Th1 and Th17 **(D)**, DC **(E)** from PBMC (n=3-6). (**F,** **G)** Flow cytometry quantification of Th1 and Th17 **(F)**, DC **(G)** from iLN (n=3-5). ns, not significant. *P < 0.05, **P < 0.01, ***P < 0.001, ****P < 0.0001 by one-way ANOVA (D-G). Data are shown as mean ± SEM.


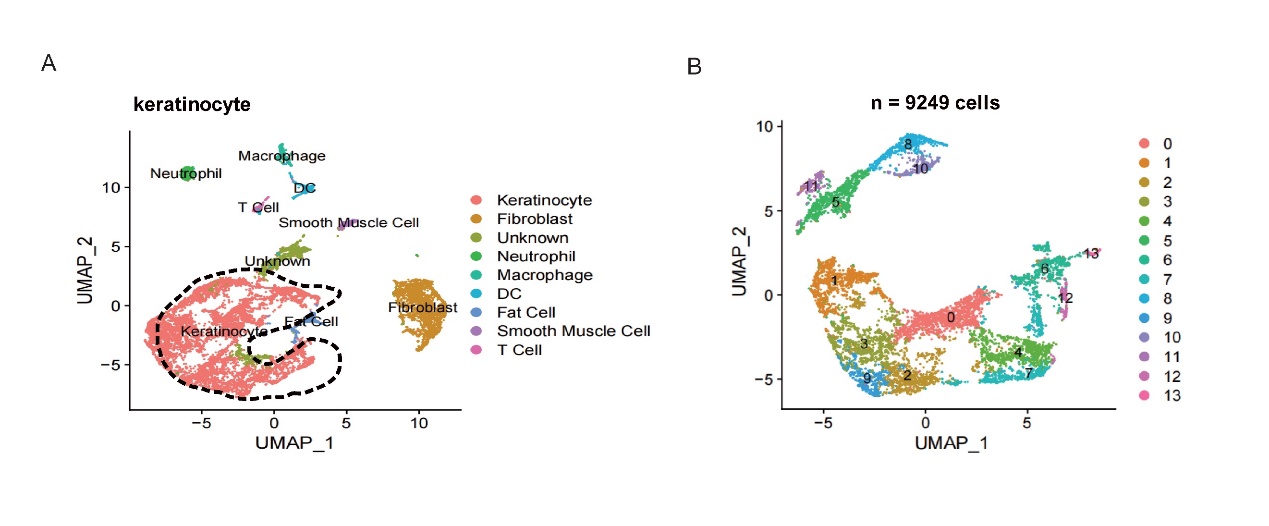


**Figure S4. Identification of KC sustypes. (A)** The UMAP plot of major cell types. **(B)** UMAP plot of KC original subclusters.

**
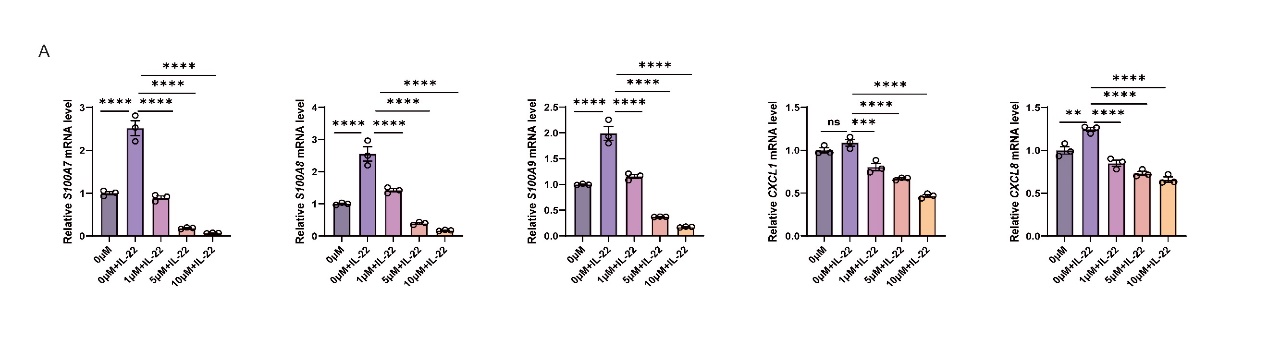
**

**Figure S5. Treatment with TYK2 inhibitor decreased the IL-22-induced proinflammatory genes in KCs. (A)** qPCR analysis of several psoriasis-associated antimicrobial peptides and chemokines in HaCaT cells after treatment with BMS-986165 in combination with IL-22 (50 ng/ml) for 48 hours (n=3). ns, not significant. **P < 0.01, ***P < 0.001, ****P < 0.0001 by one-way ANOVA (A). Data are shown as mean ± SEM.


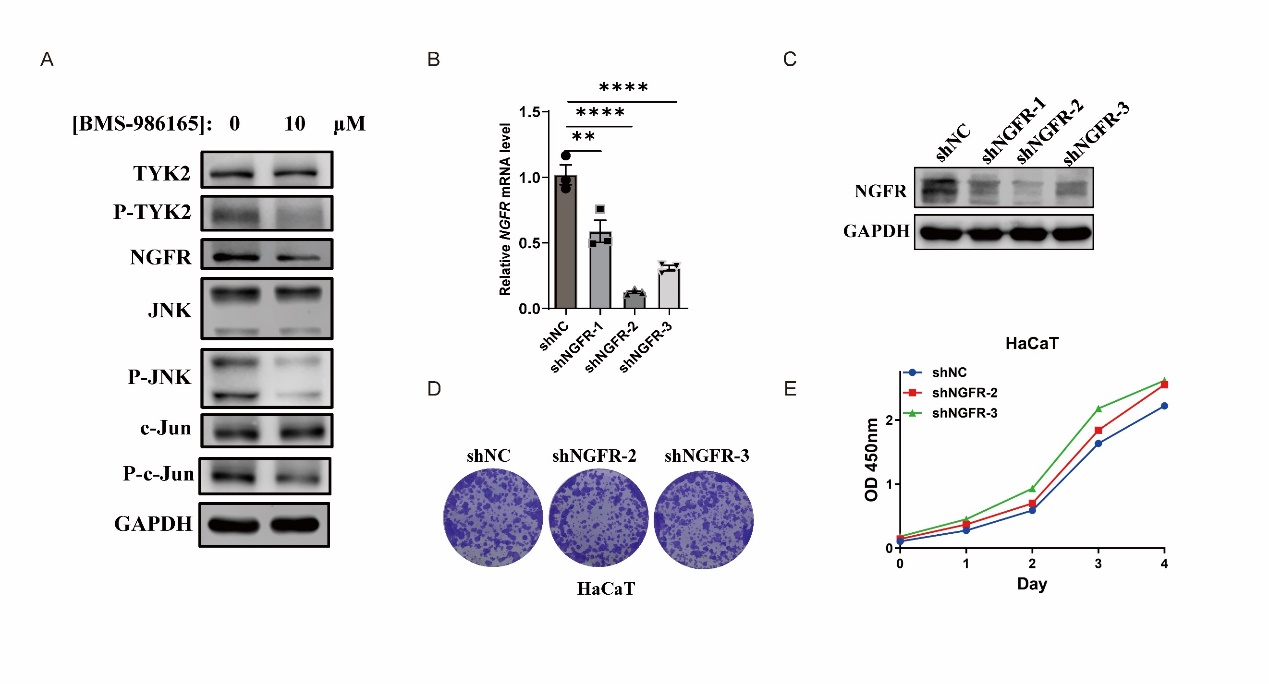


**Figure S6. NGFR knockdown did not impact the proliferation of KCs. (A)** Western blot analysis of specified protein expression in primary KCs (NHEK) with or without BMS-986165 for 48 hours. **(B, C)** qPCR **(B)** (n=3) and western blot **(C)** analysis on expression of NGFR in HaCaT cells after NGFR silencing by three different sequences of shNGFR. **(D, E)** The proliferative ability of HaCaT cells after NGFR knockdown was evaluated by colony formation assay **(D)** and CCK-8 assay **(E)**. **P < 0.01, ****P < 0.0001 by one-way ANOVA (A). Data are shown as mean ± SEM.


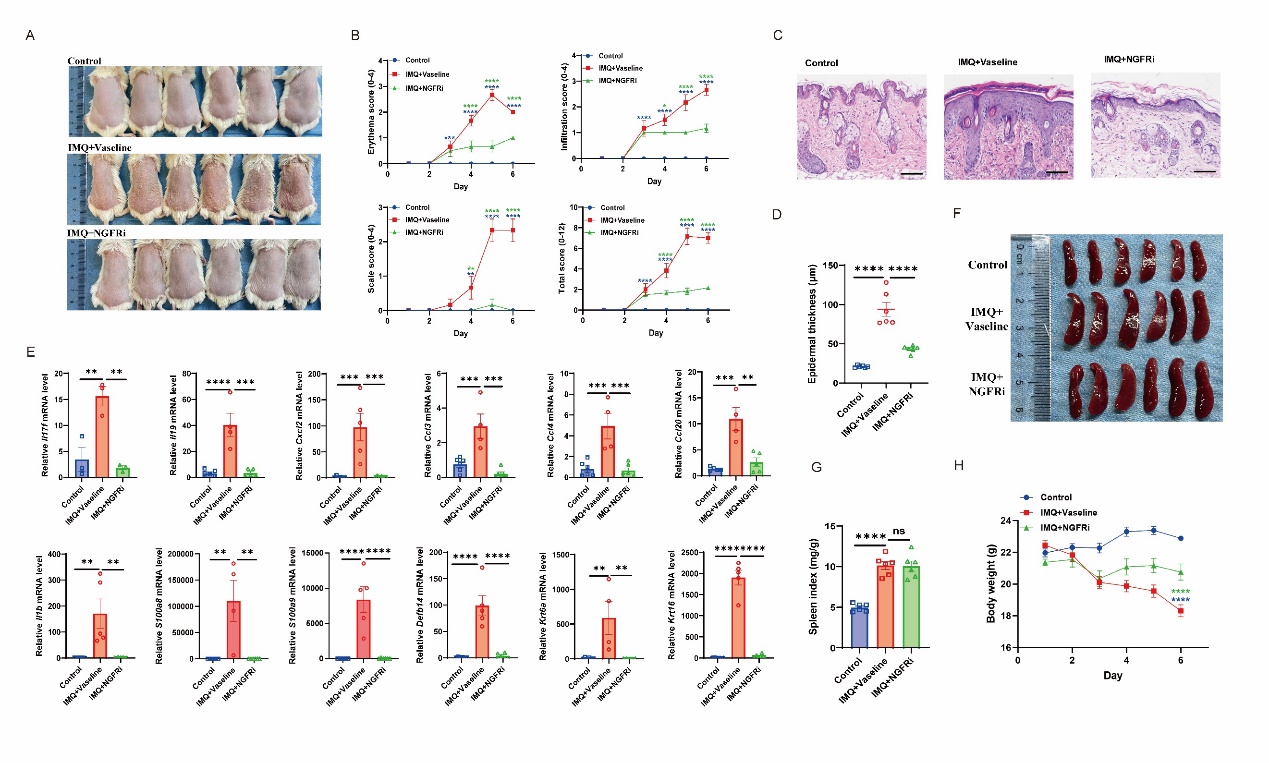


**Figure S7. Topical application of NGFR antagonist effectively mitigated the IMQ-induced psoriatic dermatitis without affecting the growth state of mice. (A)** Macroscopic images for control and psoriatic mice either topically treated with Vaseline or 1.5% NGFR antagonist THX-B (NGFRi) ointment (n=6). **(B)** Disease severity score (erythema, infiltration, scale, and total score) (n=6). **(C, D)** Representative images of H&E staining in mouse lesional skin **(C)** and quantification on epidermal thickness **(D)** (n=6). Scale bars, 100 μm. **(E)** qPCR analysis on mRNA expression of several psoriasis-related genes in total skin lesions from mice (n=3-6). **(F)** The macroscopic images of spleen. **(G)** The spleen index of mice (n=6). **(H)** The change in body weight of mice during psoriasis model induction (NGFRi, NGFR antagonist THX-B) (n=6). ns, not significant. ***P* < 0.01, ****P*<0.001, ****P < 0.0001 by one-way ANOVA **(D, E, G)** and two-way ANOVA **(B, H)**. Data are shown as mean ± SEM.

**Table S1** **qPCR primer nucleotide sequences table**

| **Gene** | **Species** | **Forward primer** | **Reverse primer** |
| --- | --- | --- | --- |
| Gapdh | mouse | AGGTCGGTGTGAACGGATTTG | TGTAGACCATGTAGTTGAGGTCA |
| S100a8 | mouse | AGTGTCCTCAGTTTGTGCAG | ACTCCTTGTGGCTGTCTTTG |
| S100a9 | mouse | ATACTCTAGGAAGGAAGGACACC | TCCATGATGTCATTTATGAGGGC |
| Il17f | mouse | TGCTACTGTTGATGTTGGGAC | AATGCCCTGGTTTTGGTTGAA |
| Il19 | mouse | CTCCTGGGCATGACGTTGATT | GCATGGCTCTCTTGATCTCGT |
| Il1b | mouse | ACGGACCCCAAAAGATGAAG | TTCTCCACAGCCACAATGAG |
| Cxcl2 | mouse | CCAACCACCAGGCTACAGG | GCGTCACACTCAAGCTCTG |
| Ccl3 | mouse | TTCTCTGTACCATGACACTCTGC | CGTGGAATCTTCCGGCTGTAG |
| Ccl4 | mouse | TTCCTGCTGTTTCTCTTACACCT | CTGTCTGCCTCTTTTGGTCAG |
| Krt16 | mouse | GGTGGCCTCTAACAGTGATCT | TGCATACAGTATCTGCCTTTGG |
| Krt6a | mouse | AGAGAGGGGTCGCATGAACT | TCATCTGTTAGACTGTCTGCCTT |
| Krt6b | mouse | CCATCAAGAGTCAAACTAGCCAC | GCTCAAACATAGGCTCCAGGTT |
| Defb4 | mouse | TTCTCCTGGTGCTGCTGTCTCC | TGCCAATCTGTCGAAAAGCGGTAG |
| Ccl20 | mouse | AAGACAGATGGCCGATGAAG | TCTTGACTCTTAGGCTGAGGA |
| Defb14 | mouse | TCCAGGGGACGCATTCCTA | ACCGCTATTAGAACATCGACCTA |
| GAPDH | human | GGAGCGAGATCCCTCCAAAAT | GGCTGTTGTCATACTTCTCATGG |
| TYK2 | human | CCTCCTGGAGATCTGCTTTG | TCTGGGTTGGCTCATAGGTC |
| S100A7 | human | CCCAACTTCCTTAGTGCCTGTGAC | GCTCTGCTTGTGGTAGTCTGTGG |
| S100A8 | human | TTGCTAGAGACCGAGTGTCCTCAG | GCCACGCCCATCTTTATCACCAG |
| S100A9 | human | GAACACATCATGGAGGACCTGGAC | GGTTAGCCTCGCCATCAGCATG |
| CXCL1 | human | CCGAAGTCATAGCCACACTCAAG | GTTGGATTTGTCACTGTTCAGCATC |
| CXCL3 | human | CCAAACCGAAGTCATAGCCAC | TGCTCCCCTTGTTCAGTATCT |
| CXCL8 | human | TTTTGCCAAGGAGTGCTAAAGA | AACCCTCTGCACCCAGTTTTC |
| STAT3 | human | ACCAGCAGTATAGCCGCTTC | GCCACAATCCGGGCAATCT |
| NGFR | human | CGTATTCCGACGAGGCCAAC | GTGTAATCCAACGGCCAGGG |
